# *psiTPTE22-HERV* functions as a tumor suppressor by inhibiting PI3K/AKT/mTOR/EIF4E signaling

**DOI:** 10.1016/j.gendis.2025.101915

**Published:** 2025-10-31

**Authors:** Fei Xu, Mengwen Zhang, Suzhan Zhang, Yao Zeng, Shu Zheng, Jessie Qiaoyi Liang

**Affiliations:** a Key Laboratory of Cancer Prevention and Intervention, China National Ministry of Education, Key Laboratory of Molecular Biology in Medical Sciences, Cancer Institute, The Second Affiliated Hospital, Zhejiang University School of Medicine, Hangzhou, Zhejiang 310009, China; b Department of Medicine and Therapeutics, Faculty of Medicine, Li Ka Shing Institute of Health Science, The Chinese University of Hong Kong, Hong Kong 999077, China; c Shenzhen Research Institute, The Chinese University of Hong Kong, Shenzhen, Guangdong 518063, China

**Keywords:** Gastric cancer, PI3K/AKT/mTOR, Prognosis, *psiTPTE22-HERV*, Tumor suppressor

## Abstract

*psiTPTE22-HERV* is a human-specific gene with unexplored functions. This study investigated its regulation, molecular mechanism, and clinical significance in gastric cancer. *psiTPTE22-HERV* expression was consistently down-regulated in primary tumors and cancer cell lines across various cancer types, including gastric cancer, as indicated by TCGA data and quantitative PCR analysis. Its down-regulation in gastric cancer cells is mediated by promoter methylation, confirmed through bisulfite genomic sequencing, with expression restored following demethylation treatment. A progressive reduction in *psiTPTE22-HERV* expression was observed from normal stomach mucosa to adjacent non-tumor tissues and primary tumors, along with increased methylation. *psiTPTE22-HERV* inhibited cancer cell viability, clonogenicity, cell cycle progression, migration, and invasion while promoting apoptosis. It also suppressed subcutaneous tumor growth and distant metastasis in mice. Transcriptomic analysis revealed that *psiTPTE22-HERV* disrupted PI3K/AKT/mTOR signaling. Western blotting confirmed that *psiTPTE22-HERV* reduced protein levels of PI3K, p-AKT, and mTOR. This led to the down-regulation of EIF4E and EIF4EBP1, oncogenic protein synthesis genes and effectors of mTOR signaling, as well as p-EIF4EBP1(Thr70), which is regulated by mTOR. Notably, *psiTPTE22-HERV* expression was inversely correlated with EIF4E, EIF4EBP1, and p-EIF4EBP1 (Thr70) in gastric tumors (all *P* < 0.001). Multivariate analysis revealed that *psiTPTE22-HERV* expression in primary gastric tumors independently predicted survival (*P* = 0.027). Kaplan–Meier survival analysis showed that low *psiTPTE22-HERV* expression was associated with shortened survival in gastric cancer patients (*P* = 0.0009). Overall, *psiTPTE22-HERV* plays a tumor-suppressive role by inhibiting the PI3K/AKT/mTOR/EIF4E pathway, and its down-regulation serves as an independent marker for poor prognosis in gastric cancer patients.

## Introduction

Gastric cancer is a prevalent malignancy and a leading cause of cancer-related mortality globally.^1^ Promoter hypermethylation-induced inactivation of tumor suppressor genes, such as *PTEN*, *MDGA2*, *ZNF331*, *REC8*, and *RASSF10*, has been implicated in the development of gastric cancer.^2^, ^3^, ^4^, ^5^, ^6^ Silencing of tumor suppressors holds diagnostic and prognostic value for gastric cancer patients. Human endogenous retroviruses (HERVs) are remnants of ancient germ line retrovirus infections that date back over a million years.^7^ HERV integrations can give rise to novel human genes that differ from their non-human counterparts or enable tissue-specific expression of HERV-related genes.^8^^,^^9^ *psiTPTE22-HERV* represents a recently evolved gene that results from the fusion of HERV and adjacent non-HERV DNA. Notably, the HERV involved is exclusive to humans, rendering *psiTPTE22-HERV* a human-specific gene.^10^ Based on a limited sample size, *psiTPTE22-HERV* was detected in the normal kidney, liver, stomach, and lung tissues, whereas its expression was diminished in the corresponding primary tumor tissues. Bisulfite genomic sequencing confirmed the suppression of *psiTPTE22-HERV* in kidney cancer owing to promoter DNA methylation. However, as a newly identified gene, the regulatory mechanisms of *psiTPTE22-HERV* in other cancers and its functional roles remain to be investigated.

The regulatory patterns, functional significance, and mechanisms of *psiTPTE22-HERV* in various cancers, including gastric cancer, require further exploration. In this study, we investigated the expression, epigenetic regulation, biological functions, molecular mechanisms, and clinical implications of *psiTPTE22-HERV* in the context of gastric cancer.

## Material and methods

### The Cancer Genome Atlas (TCGA) data

Transcript levels of *psiTPTE22-HERV* were obtained from TSVdb,^11^ a Web tool for splicing variant analysis in the TCGA dataset (http://www.tsvdb.com). The specific isoforms used in the analysis were isoform_uc010gqq and isoform_uc002zlr, which correspond to the two open-reading-frame-containing transcript variants of *psiTPTE22-HERV*. In addition, protein data (reverse-phase protein array) for eukaryotic translation initiation factor 4E (EIF4E) binding protein 1 (EIF4EBP1), EIF4EBP1-pT70, and EIF4E were acquired from the Stomach Adenocarcinoma dataset of TCGA (Firehose Legacy) through the cBioPortal for Cancer Genomics (http://www.cbioportal.org/).^12^^,^^13^

### Cancer cell lines

Cancer cell lines (MKN45, SGC7901, and MKN1) were grown in 1640 medium supplemented with 10% fetal bovine serum and 1% penicillin–streptomycin at 37 °C in a humidified 5% CO_2_ atmosphere. MKN45 and SGC7901 cells were obtained from Beyotime (Shanghai, China), with subsequent identification of potential contamination with HeLa cells in SGC7901. MKN1 cells were obtained from RIKEN BioResource Research Center (Tsukuba, Japan). All experiments were performed using mycoplasma-free cells. cDNAs and/or DNA of additional cancer cell lines and the human embryonic kidney HEK293 cell line were obtained from previous studies^3^^,^^4^ or from stocks at the Cancer Institute of Zhejiang University. A complete list of the cell lines used in this study is provided in Table S1.

### Primary tissue samples

Gastric cancer tissues were collected during surgical resection at the Second Affiliated Hospital of Zhejiang University in Hangzhou, China. This study adhered to the principles outlined in the Helsinki Declaration. This study was approved by the Clinical Research Ethics Committee of Zhejiang University (IRB# 2016-133). Written informed consent was obtained from all participants. cDNAs of normal human tissues were purchased from Stratagene (La Jolla, California, USA).

### RNA extraction and PCR

Total RNA was extracted from cells and tissues using TRIzol reagent (Invitrogen, Carlsbad, California, USA), according to the manufacturer's instructions. cDNA was synthesized using the One Step PrimeScript cDNA Synthesis Kit (TaKaRa, Dalian, China). Reverse transcription PCR was performed using the GoTaq Colorless Master Mix (Promega, Madison, Wisconsin, USA). Quantitative PCR was performed using the AceQ qPCR Probe Master Mix (Vazyme Biotech, Nanjing, China). The nucleotide sequences of PCR primers and probes are listed in Table S2. Each probe carried a 5′ reporter dye, 6-carboxy fluorescein (FAM), and a 3′ quencher dye, 6-carboxytetramethyl-rhodamine (TAMRA). Primers and hydrolysis probes were synthesized by Invitrogen. Quantitative PCR amplifications were conducted in a 20 μL reaction system containing 0.3 μM of each primer and 0.2 μM of probe. The thermal cycler parameters of the ABI PRISM 7500 Fast Sequence Detection System were 50 °C for 2 min, 95 °C for 10 min, and 40 cycles of 95 °C for 15 s and 60 °C for 1 min. PCR amplification specificity was confirmed by Sanger sequencing of the PCR products. The relative expression level of *psiTPTE22-HERV* was calculated using the ΔCt method relative to that of GAPDH.

### Bisulfite genomic sequencing

Bisulfite modification was performed using the EZ DNA methylation kit (Zymo Research, Orange, California, USA) following the manufacturer's instructions. PCR amplification was performed using 2 μL bisulfite-converted DNA. Direct Sanger sequencing of the PCR products was performed to evaluate the methylation levels. The proportion of methylation was calculated as the peak ratio of cytosine to the sum of cytosine and thymine at each site. The nucleotide sequences of the primers used for bisulfite genomic sequencing are listed in Table S2.

### Demethylation with 5-Aza agent treatment

Gastric cancer cells were treated with 2 μM DNA demethylation agent, 5-Aza (Sigma–Aldrich, St Louis, Missouri, USA), for 5 days. The culture medium was refreshed every day.

### Establishment of stable *psiTPTE22-HERV-*expressing cells

The full-length open reading frame of *psiTPTE22-HERV* was generated by reverse transcription PCR and cloned into the mammalian expression vector pcDNA™3.1/myc-His A (Invitrogen). The construct sequence was confirmed by sequencing. MKN45 and SGC7901 cells were transfected with pcDNA3.1 or pcDNA3.1-*psiTPTE22-HERV* plasmids using Lipofectamine 3000 (Invitrogen). Stable transfectants were selected with G418 antibiotic (Invitrogen) for two weeks.

### Knockdown of *psiTPTE22-HERV* by RNA interference

Knockdown of *psiTPTE22-HERV* expression in MKN1 cells was performed using RNA interference with a small interfering RNA (siRNA) specifically targeting *psiTPTE22-HERV* (Table S2). siRNA was transfected using Lipofectamine 3000, with a scrambled sequence as the small interfering negative control RNA (siNC). Knockdown efficiency was evaluated using reverse transcription or quantitative PCR.

### Cell viability and colony formation

Cell viability was assessed using the CCK-8 assay (Boster Bio, Pleasanton, California, USA). For colony formation, cells were seeded at a density of 200 cells per well in 6-well culture plates and incubated at 37 °C for at least 14 days until visible colonies formed. The colonies were fixed with methanol for 15 min and stained with 0.5% crystal violet. Colonies consisting of > 50 cells were counted.

### Cell cycle analysis

Cells were processed using a cell cycle kit from MultiSciences Biotech (Hangzhou, China) following the manufacturer's instructions. The cells were then sorted using a FACS Calibur Flow Cytometer (BD Biosciences, Franklin Lakes, New Jersey, USA). Cell cycle distribution was analyzed using the ModFitLT software (BD Biosciences).

### Apoptosis

Cell apoptosis was assessed using Annexin V-APC/7-AAD staining (MultiSciences Biotech) according to the manufacturer's instructions, followed by flow cytometry. Additionally, in tumor biopsies from nude mice, apoptosis was measured using the terminal deoxynucleotidyl transferase-mediated dUTP-digoxigenin nick-end labelling (TUNEL) assay (Promega). Nuclei with clear staining were regarded as TUNEL-positive apoptotic cells. The apoptosis index was calculated as the percentage of TUNEL-positive nuclei, with at least 1000 cells counted.

### Wound-healing and invasion assays

For wound-healing assays, cells were seeded in 6-well culture plates and allowed to reach 80% confluence after 24 h. Wounds were created using 200-μL plastic pipette tips, followed by washing with phosphate-buffered saline (PBS). The wounded cell monolayers were then allowed to heal for 48 h or 72 h in serum-free RPMI 1640 medium. Invasion assays were conducted using Matrigel™ Invasion Chambers (BD Biosciences). Cells suspended in serum-free medium (1 × 10^5^ cells in 100 μL) were added to the upper chamber, whereas medium containing 10% fetal bovine serum was added to the lower chamber. After incubation at 37 °C for 48 h, the number of cells that migrated to the lower side of the upper chamber was determined.

### *In vivo* subcutaneous tumorigenicity and metastasis assays

All animal experiments were conducted in accordance with institutional guidelines and were approved by the Animal Experimental Committee of Zhejiang University (#2016-239). For subcutaneous xenograft models, MKN45 cells (5 × 10^6^ cells in 200 μL PBS) stably transfected with *psiTPTE22-HERV* expression vector or empty vector were injected subcutaneously into the backs of 5-week-old male Balb/c nude mice (control: *n* = 10; *psiTPTE22-HERV*: *n* = 11). Tumor diameter was measured every 3 days for 30 days, and the volume (mm^3^) was calculated as follows: (shortest diameter)^2^ × (longest diameter) × 0.5. Upon sacrifice, the tumors were excised, weighed, and either fixed in 10% neutral-buffered formalin or snap-frozen in liquid nitrogen. For tail vein injection models, SGC7901 cells stably transfected with *psiTPTE22-HERV* expression vector or empty vector (2 × 10^6^ cells in 100 μL PBS) were injected into the tail veins of 6-week-old female Balb/c nude mice (control: *n* = 10; *psiTPTE22-HERV*: *n* = 11). Four weeks after the injection, the mice were sacrificed and examined. The lungs and livers were dissected, paraffin-embedded, and sectioned. Hematoxylin-eosin staining was performed, and the metastatic tumors were counted in a blinded manner. No animals exhibited significant discomfort, impaired mobility, or compromised well-being during the study period.

### Western blotting and immunohistochemistry

Total cellular proteins were extracted with a RIPA lysis and extraction buffer. Fifty micrograms of protein from each sample were separated on a 10% SDS-PAGE gel and then transferred onto nitrocellulose membranes (GE Healthcare). After incubation with specific primary antibodies at 4 °C overnight and then with secondary antibodies, protein bands were visualized using ECL Plus Western Blotting Detection Reagents (GE Healthcare). Immunohistochemistry was conducted on 5-μm paraffin-embedded xenograft sections. The staining intensity was evaluated as the percentage of cells that displayed positive staining. The antibodies used in this study are listed in Table S3.

### Immunofluorescence assay

The cells were fixed with 4% paraformaldehyde in PBS on slides at room temperature for 30 min. The cells were permeabilized using 0.2% Triton X-100 in PBS (pH 7.2) for 4 min and blocked with 5% bovine serum albumin in PBS at room temperature for 30 min. Cells were stained with FITC-conjugated mouse anti-EIF4E antibody (1:100 dilution in PBS containing 1% bovine serum albumin; BD Biosciences) at 4 °C overnight and then with Dylight 488-conjugated goat anti-mouse polyclonal antibody (Abcam) at room temperature for 1 h. After three PBS washes (pH 7.2), the slides were mounted using Vectashield containing DAPI (Vector Laboratories, Burlingame, California, USA) and sealed. Fluorescence was observed using a Zeiss Confocal Laser Scanning Microscope.

### RNA sequencing and differential gene expression

Total RNA (3 μg) with RNA integrity number > 6.8 from cells stably transfected with either *psiTPTE22-HERV* expression vector or empty vector was subjected to RNA sequencing. The mRNA was isolated using Oligo d(T) Magnetic Beads and fragmented for cDNA synthesis. Libraries were prepared using the NEBNext Ultra™ RNA Library Prep Kit (New England Biolabs, Ipswich, Massachusetts, USA) following the manufacturer's instructions and sequenced on an Illumina HiSeq XTEN platform. Raw sequencing reads were quality filtered to remove adapter sequences, poly-N reads (> 10% N content), and low-quality reads (Q-score < 20). All samples yielded > 3.25 Gb clean bases with a < 0.02% error rate and Q20 of > 96.4%. Clean reads were aligned to the reference genome using HISAT2,^14^ and gene expression levels were quantified as FPKM (Fragments Per Kilobase of transcript per Million mapped reads) and read counts using FeatureCounts v1.5.0-p3.^15^ Differential expression analysis was performed using the edgeR R package. The resulting *P*-values were adjusted using the Benjamini and Hochberg method for multiple testing.

### Over-representation analysis and gene set enrichment analysis (GSEA)

Enrichment analysis was performed on differentially expressed genes induced by *psiTPTE22-HERV* overexpression to identify significant gene ontology (GO) biological processes and Kyoto Encyclopedia of Genes and Genomes (KEGG) pathways. Differentially expressed genes were subjected to over-representation analysis and GSEA using the WebGestalt platform (http://www.webgestalt.org/#).^16^ GO analysis was performed using the Biological Process noRedundant database with default parameters.

### Statistical analysis

Data were shown as mean ± standard deviation, mean ± standard error of the mean, or median (interquartile range), as appropriate. All functional data were obtained from at least three independent experiments. The Mann–Whitney *U* test was performed for comparisons between two sample groups. Percentages were compared between two groups using Fisher's exact test. Differences between experimental groups were assessed using Student's *t*-test or one-way ANOVA. Linear trends in mRNA expression and promoter methylation were analyzed using one-way ANOVA with multiple comparison tests. The cutoff value was determined by survival significance analysis using Cutoff Finder (http://molpath.charite.de/cutoff/).^17^ Kaplan–Meier analysis with the log-rank test was used to compare survival distributions between two groups. Hazard ratios were estimated using univariate and multivariate Cox regression analyses to assess the association between predictor variables and risk of death. The difference in tumor growth between the two groups of nude mice was determined using repeated-measures ANOVA. All statistical tests were conducted using SPSS 19.0 software (SPSS Inc., Chicago, Illinois, USA) and GraphPad Prism V9.4 (GraphPad Software, Inc., San Diego, California, USA). *P* values < 0.05 were considered statistically significant.

## Results

### *psiTPTE22-HERV* is commonly down-regulated in primary tumors across various cancer types

**Figure 1:**
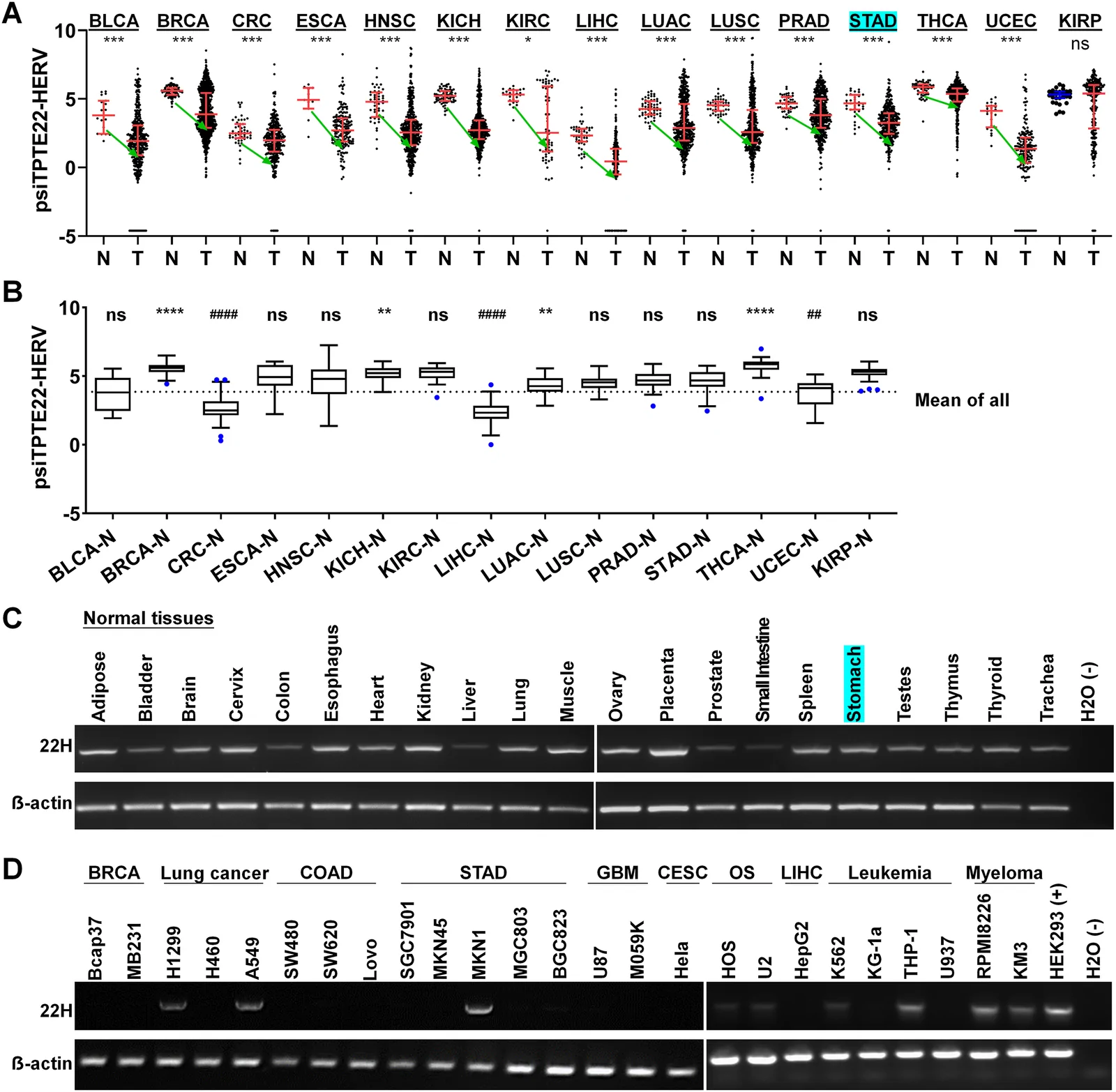
Down-regulation of *psiTPTE22-HERV* in tumor tissues across multiple cancer types. **(A)** Significant down-regulation of *psiTPTE22-HERV* in primary tumors versus matched non-tumor tissues in 14/15 cancer types. **(B)** Robust *psiTPTE22-HERV* expression in non-tumor tissues. **(C, D)***psiTPTE22-HERV* expression in normal adult tissues, including stomach mucosa, and its silencing/down-regulation in cancer cell lines as indicated by PCR. The data were presented as median (interquartile range) (A) or box-and-whiskers (Tukey) (B) and compared using Mann–Whitney *U* test (A) or Kruskal–Wallis test (B; versus all data). ∗*P <* 0.05, ∗∗*P <* 0.01, ∗∗∗*P <* 0.001, and ∗∗∗∗*P <* 0.0001.

To comprehensively evaluate *psiTPTE22-HERV* expression in cancers, we analyzed the TCGA data. Among the 15 cancer types with both tumor and adjacent non-tumor tissues, *psiTPTE22-HERV* was significantly down-regulated in tumors compared with non-tumor tissues in 14 types, including gastric cancer (Fig. 1A). The exception was kidney renal papillary cell carcinoma, which showed no substantial down-regulation. Most non-tumor tissues displayed ubiquitous *psiTPTE22-HERV* expression, with 12 out of 15 types showing similar or higher levels relative to all non-tumor tissues (Fig. 1B). Reverse transcription PCR confirmed *psiTPTE22-HERV* expression in various normal adult tissues, including the normal stomach (Fig. 1C), whereas its expression was not detected in 76% (19/25) of the cancer cell lines (Fig. 1D). These findings demonstrate that *psiTPTE22-HERV* is expressed in most normal tissues and is consistently down-regulated across a wide spectrum of cancer types.

### *psiTPTE22-HERV* is down-regulated by promoter methylation in gastric cancer cells and primary tumors

**Figure 2:**
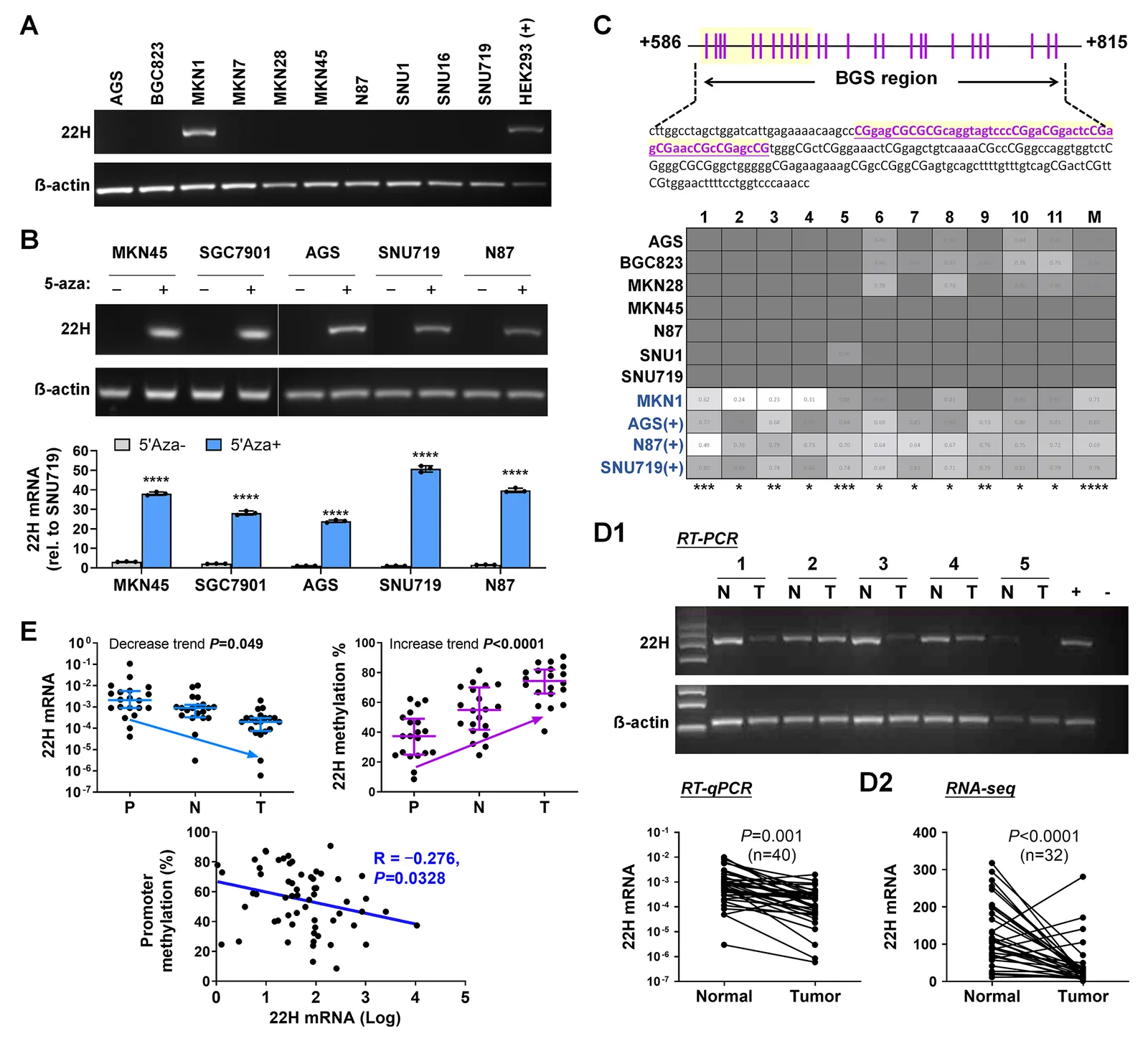
Regulation of *psiTPTE22-HERV* expression by promoter DNA methylation. **(A)** Silencing of *psiTPTE22-HERV* in 9/10 gastric cancer cell lines. **(B)** Restoration of *psiTPTE22-HERV* expression by 5-Aza demethylation treatment in gastric cancer cell lines. **(C)** Bisulfite genomic sequencing revealed hypermethylation of *psiTPTE22-HERV* promoter (11 CpG sites, underlined) in silenced gastric cancer cells, with reduced methylation in MKN1 and 5-Aza treated cells. **(D)** Down-regulation of *psiTPTE22-HERV* in primary gastric tumors versus paired normal tissues by PCR (D1; in-house samples) and RNA sequencing (D2; TCGA). **(E)** Progressive decrease in expression and increase in promoter methylation, observed from normal stomach mucosa to adjacent non-tumor tissues to primary tumors, along with a significant inverse correlation between expression and promoter methylation in all samples. The data were presented as mean ± standard deviation (B), mean (C), or median (interquartile range) (E) and compared using an unpaired *t*-test (B, C) or one-way ANOVA (E). ∗*P <* 0.05, ∗∗*P <* 0.01, ∗∗∗*P <* 0.001, and ∗∗∗∗*P <* 0.0001.

We further investigated the expression and regulatory mechanism of *psiTPTE22-HERV* in gastric cancer. *psiTPTE22-HERV* was silenced in the majority of the tested gastric cancer cell lines (9/10, 90%) (Fig. 2A), but its expression was restored following treatment with the DNA demethylation agent 5-Aza (Fig. 2B). The bisulfite genomic sequencing results demonstrated that cells with down-regulated or silenced *psiTPTE22-HERV* exhibited significantly higher methylation levels at 11 out of the 26 CpG sites analyzed than cells with *psiTPTE22-HERV* expression or restoration (*P* < 0.05; Fig. 2C). The PCR analyses revealed that *psiTPTE22-HERV* expression was significantly down-regulated in gastric tumors compared with paired adjacent non-tumor tissues in Chinese patients (*n* = 40; *P* = 0.001; Fig. 2D1). This finding was corroborated by RNA-sequencing data from TCGA (*n* = 32; *P* < 0.0001; Fig. 2D2). Furthermore, *psiTPTE22-HERV* expression showed a significant decreasing trend from distant normal stomach mucosa to adjacent non-tumor tissues and further to primary tumors (*P* < 0.05). Conversely, promoter methylation levels exhibited a significant increasing trend (*P* < 0.0001). Additionally, there was a significant inverse correlation between *psiTPTE22-HERV* expression and promoter methylation in these samples (*r* = −0.276; *P* = 0.0328; Fig. 2E). These findings indicate that aberrant methylation at specific CpG sites in the *psiTPTE22-HERV* promoter region contributes to its down-regulation in gastric cancer.

### *psiTPTE22-HERV* plays a tumor-suppressive role *in vitro*

**Figure 3:**
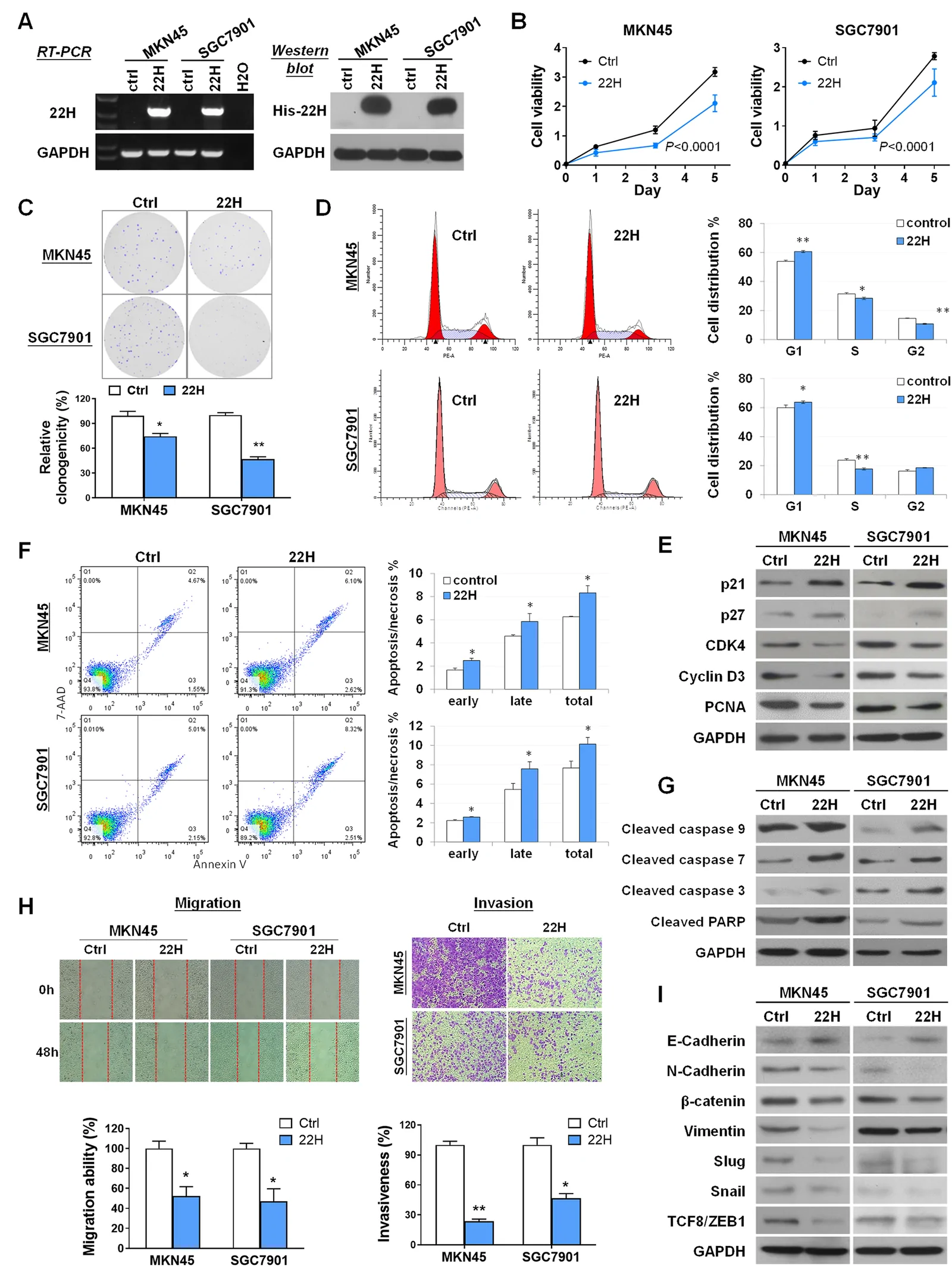
*psiTPTE22-HERV* exerts tumor-suppressive effects *in vitro*. **(A)** Ectopic expression of *psiTPTE22-HERV* occurred in MKN45 and SGC7901 cells, as confirmed by PCR and western blotting. **(B)** Overexpression of *psiTPTE22-HERV* significantly inhibited cell viability of cancer cells. **(C)** Overexpression of *psiTPTE22-HERV* significantly inhibited colony formation of cancer cells. **(D)** Overexpression of *psiTPTE22-HERV* caused G1–S cell cycle arrest as indicated by flow cytometry. **(E)** Overexpression of *psiTPTE22-HERV* altered the expression of cell cycle-related proteins as revealed by western blotting. **(F)** Overexpression of *psiTPTE22-HERV* induced apoptosis/necrosis of cancer cells as indicated by flow cytometry. **(G)** Overexpression of *psiTPTE22-HERV* induced activation of apoptotic proteins. **(H)** Overexpression of *psiTPTE22-HERV* suppressed cell migration and invasion, as shown by wound healing and Matrigel invasion assays, respectively. **(I)** Overexpression of *psiTPTE22-HERV* altered the expression of epithelial–mesenchymal transition markers. The data were presented as mean ± standard deviation (B–F) and compared using two-way ANOVA (B) or unpaired *t*-test (C–F). ∗*P <* 0.05 and ∗∗*P <* 0.001.

To investigate its function, *psiTPTE22-HERV* was overexpressed in two cancer cell lines (MKN45 and SGC7901) with low endogenous expression, as confirmed by reverse transcription PCR and western blotting (Fig. 3A). Overexpression of *psiTPTE22-HERV* significantly inhibited cell growth and colony formation (Fig. 3B and C). Flow cytometry analysis revealed that *psiTPTE22-HERV* expression induced G1–S phase cell cycle arrest, with an increase in the number of G1-phase cells and a concomitant decrease in the number of S-phase cells (Fig. 3D1). This arrest was further supported by the up-regulation of G1 cell cycle inhibitors (p21 and p27) and the down-regulation of key G1 regulators (CDK4 and cyclin D3). The decrease in S-phase cells was confirmed by the down-regulated proliferation marker, proliferating cell nuclear antigen (PCNA) (Fig. 3D2). Furthermore, *psiTPTE22-HERV* expression significantly induced apoptosis (Fig. 3E1), accompanied by activation of caspase-9, caspase-7, caspase-3, and poly(ADP-ribose) polymerase (PARP) (Fig. 3E2). Additionally, *psiTPTE22-HERV* markedly decreased cell migration and invasiveness, as demonstrated by the wound healing and Matrigel invasion assays (Fig. 3F1). These findings were supported by changes in epithelial–mesenchymal transition markers, including up-regulation of the epithelial marker E-cadherin and down-regulation of mesenchymal markers, including N-cadherin, β-catenin, Vimentin, Slug, Snail, and transcription factor 8 (TCF8)/zinc finger E-box binding homeobox 1 (ZEB1) (Fig. 3F2).

### Knockdown of *psiTPTE22-HERV* mitigates its tumor-suppressive role *in vitro*

**Figure 4:**
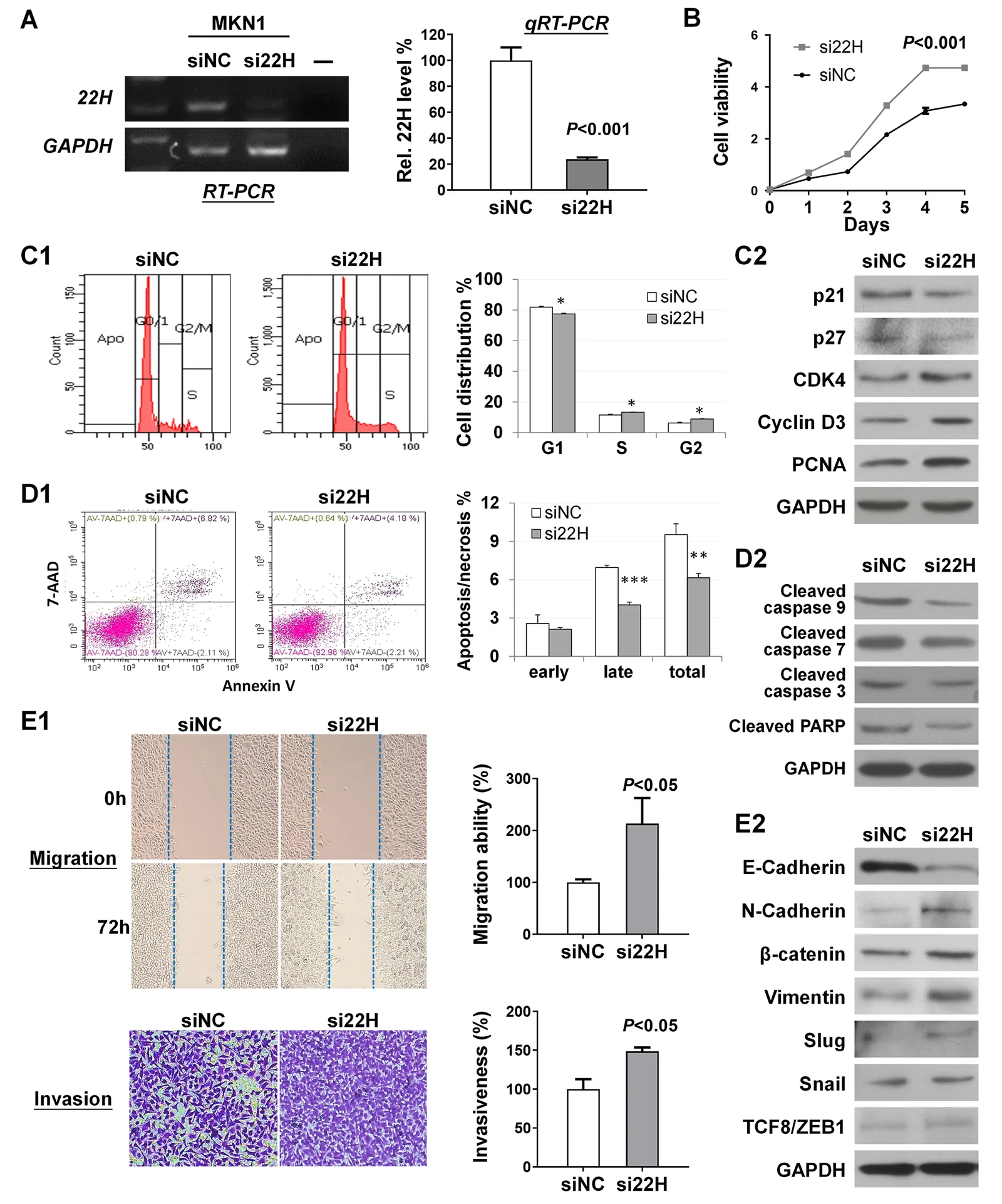
Functional consequences of *psiTPTE22-HERV* knockdown *in vitro*. **(A)** Successful siRNA-mediated knockdown in MKN1 cells confirmed by PCR. **(B)** Enhanced cell viability following *psiTPTE22-HERV* depletion. **(C)** Accelerated cell cycle progression (C1) and altered cell cycle-related protein expression (C2). **(D)** Reduced apoptosis (D1) with decreased apoptotic protein activation (D2). **(E)** Increased cell migration and invasion (E1) and altered expression of epithelial–mesenchymal transition markers (E2). The data were presented as mean ± standard deviation (B–E) and compared using two-way ANOVA (B) or unpaired *t*-test (C–E). ∗*P <* 0.05, ∗∗*P <* 0.001, and ∗∗∗*P <* 0.0001.

*psiTPTE22-HERV* was knocked down in MKN1 cells, which exhibit high endogenous expression (Fig. 4A). *psiTPTE22-HERV* knockdown significantly increased cell growth (Fig. 4B) and promoted cell cycle progression, leading to reduced cells in the G1 phase and increased cells in the S and G2 phases (Fig. 4C1). These changes were accompanied by down-regulated G1 cell cycle inhibitors (p21 and p27), elevated G1 regulators (CDK4 and cyclin D3), and increased PCNA (Fig. 4C2). Furthermore, *psiTPTE22-HERV* knockdown significantly reduced cell apoptosis (Fig. 4D1), as evidenced by decreased activation of caspase-9, caspase-7, caspase-3, and PARP (Fig. 4D2). In addition, *psiTPTE22-HERV* knockdown enhanced the migration and invasion capabilities of MKN1 cells (Fig. 4E1), along with a marked decrease in E-cadherin expression and increased expression of N-cadherin, β-catenin, Vimentin, Slug, Snail, and TCF8/ZEB1 (Fig. 4E2). These findings further underscore the tumor-suppressive role of *psiTPTE22-HERV*.

### *psiTPTE22-HERV* attenuates subcutaneous tumor growth and reduces metastasis of cancer cells in nude mice

**Figure 5:**
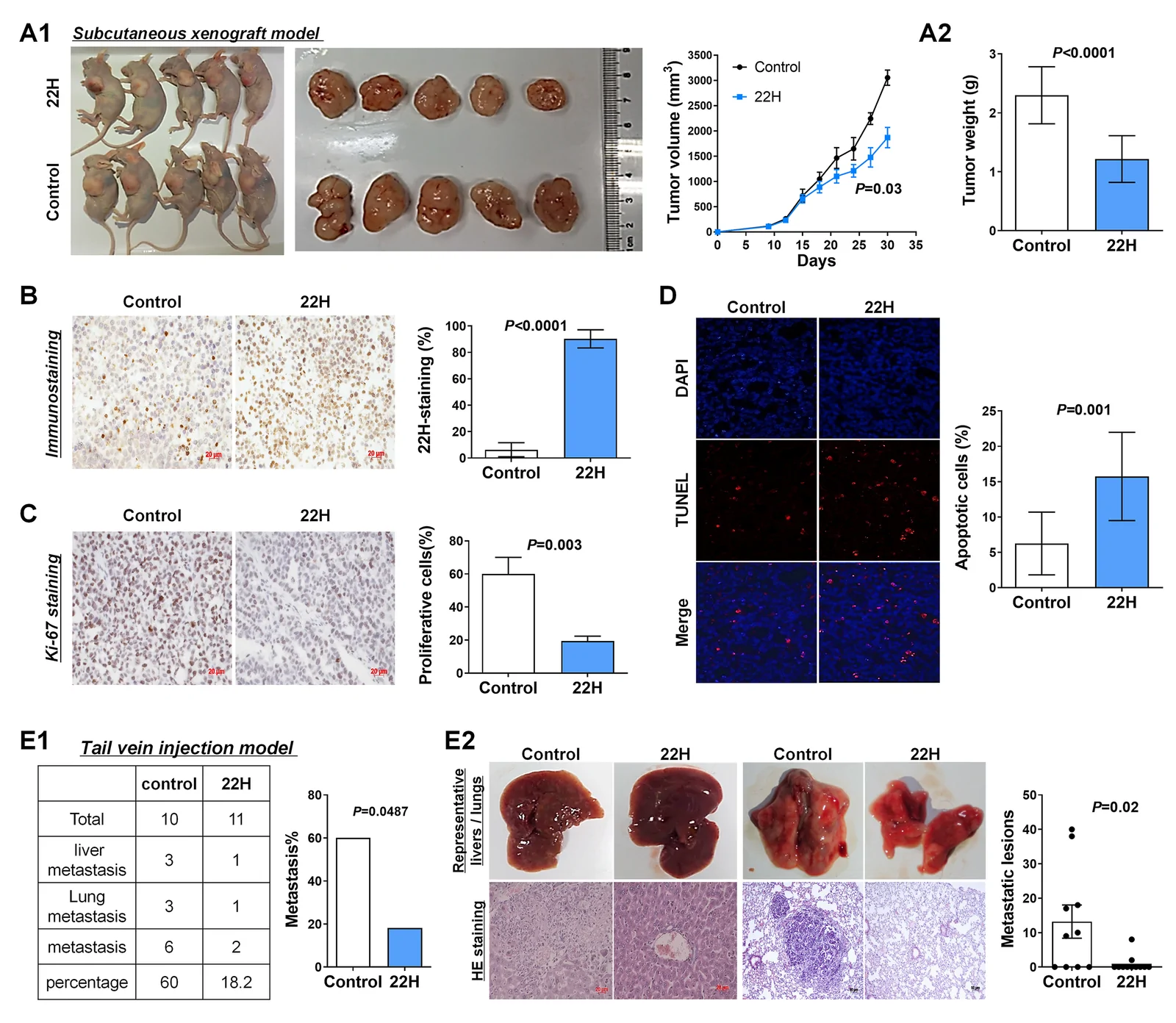
*psiTPTE22-HERV* suppressed subcutaneous tumor growth and metastasis *in vivo*. **(A)** Reduced subcutaneous tumor growth in nude mice with *psiTPTE22-HERV*-transfected cells versus controls in terms of tumor volume (A1) and tumor weight (A2). **(B)***psiTPTE22-HERV* expression in xenografts confirmed by immunostaining. **(C)** Decreased proliferation in *psiTPTE22-HERV*-expressing tumors indicated by Ki-67 staining. **(D)** Increased apoptosis in *psiTPTE22-HERV*-expressing tumors revealed by TUNEL staining. **(E)** Reduced liver and lung metastasis shown by representative macroscopic images (E1) and hematoxylin-eosin staining (E2). The data were presented as mean ± standard error of the mean (A1, A2, E2) or mean ± standard deviation (B–D) and compared using two-way ANOVA (A1), unpaired *t*-test (A2, B–D), chi-square test, or Mann–Whitney *U* test (E2).

To evaluate the effect of *psiTPTE22-HERV* on gastric tumor growth *in vivo*, subcutaneous xenograft tumor models were established by injecting MKN45 cells stably transfected with a *psiTPTE22-HERV* expression vector or an empty vector into nude mice. Tumor growth was significantly suppressed in the *psiTPTE22-HERV*-transfected group (Fig. 5A1). At the end of the experiment, the net weight of tumors in the *psiTPTE22-HERV*-transfected group was also significantly reduced (Fig. 5A2). Immunohistochemistry confirmed *psiTPTE22-HERV* expression in xenograft tumors in the *psiTPTE22-HERV*-transfected group (Fig. 5B). Consistent with the *in vitro* findings, *psiTPTE22-HERV*-expressing tumors exhibited fewer proliferating cells and more apoptotic cells, as demonstrated by the Ki-67 and TUNEL assays, respectively (Fig. 5C and D).

To assess the effect of *psiTPTE22-HERV* on tumor metastasis *in vivo*, SGC7901 cells stably transfected with *psiTPTE22-HERV* expression vector or empty vector were injected into the tail veins of female nude mice. After four weeks, metastasis to distant organs was evaluated. Histological examination revealed that 60% (6/10) of the control mice developed liver or lung metastases, compared with only 18.2% (2/11) of the mice bearing *psiTPTE22-HERV*-expressing cells (Fig. 5E1). The control group also exhibited significantly more metastatic lesions than the *psiTPTE22-HERV* group (Fig. 5E2). These results demonstrate the critical role of *psiTPTE22-HERV* in the suppression of cancer metastasis.

### *psiTPTE22-HERV* disrupts protein metabolism and mTOR signaling

**Figure 6:**
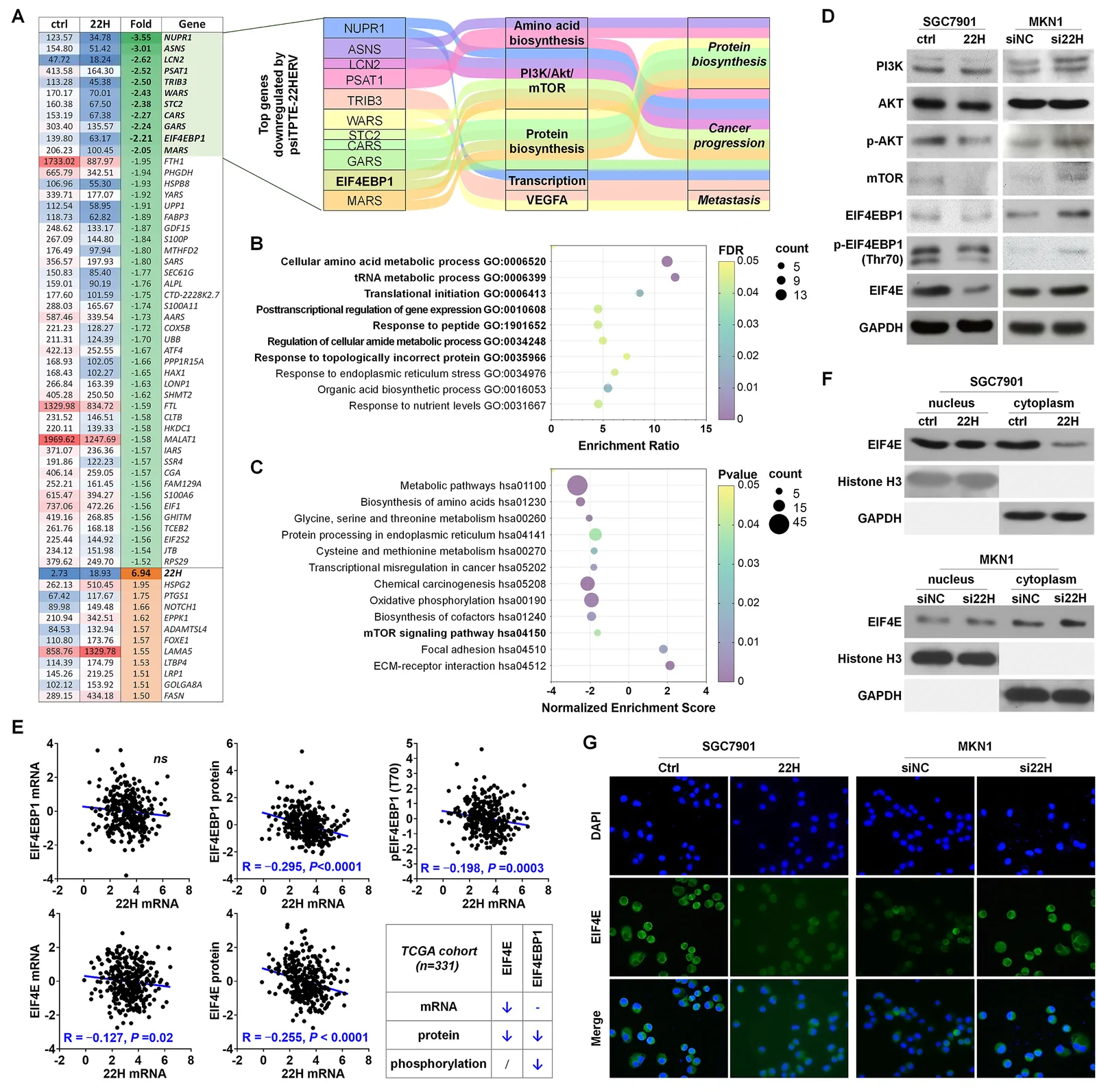
Molecular mechanisms of *psiTPTE22-HERV*-mediated tumor suppression. **(A)** Heatmap of differentially expressed genes (DEGs; |fold change| ≥ 1.5 and FDR <0.05) identified by RNA sequencing, with functional annotations for top hits (|fold change| ≥ 2). **(B)** GO biological processes altered by *psiTPTE22-HERV* overexpression as identified by over-representation analysis. **(C)** KEGG pathways enriched by DEGs as identified by GSEA. **(D)** Western blotting analysis of PI3K/AKT/mTOR pathway components (PI3K, AKT, p-AKT, and mTOR), EIF4EBP1, p-EIF4EBP1(Thr70), and EIF4E. **(E)** Pearson's correlations of *psiTPTE22-HERV* expression with EIF4EBP1/EIF4E mRNA, EIF4EBP1/EIF4E protein, and p-EIF4EBP1(Thr70) protein levels in gastric tumors of the TCGA cohort. **(F)** Cytoplasmic-specific reduction of EIF4E by *psiTPTE22-HERV*, as evidenced by western blotting of cytoplasmic and nuclear fractions. **(G)** Immunofluorescence staining confirmation of EIF4E modulation by *psiTPTE22-HERV*.

To elucidate the molecular mechanism of *psiTPTE22-HERV*, we investigated the gene expression profiles using RNA sequencing. Interestingly, the majority of the top genes down-regulated by *psiTPTE22-HERV* (|fold change| ≥ 2 and FDR < 0.05) were involved in protein synthesis and PI3K/AKT/mTOR signaling, including EIF4EBP1 (eukaryotic translation initiation factor 4E binding protein 1) (Fig. 6A). GO analysis of 60 differentially expressed genes (|fold change| ≥ 1.5, FDR < 0.05) revealed 10 significantly enriched biological processes (FDR < 0.05; ≥ 5 genes involved), 7 of which were associated with protein synthesis/metabolism (Fig. 6B; Tables S4 and S5). Further pathway enrichment analysis identified mTOR signaling to be dysregulated by *psiTPTE22-HERV*, involving the down-regulation of EIF4EBP1 (Fig. 6C; Tables S6 and S7). *EIF4EBP1* plays an oncogenic role and is a key effector of PI3K/AKT/mTOR signaling.^18^, ^19^, ^20^ Additionally, EIF4E, a direct target of *EIF4EBP1* with oncogenic roles linked to AKT and mTOR signaling,^21^^,^^22^ was down-regulated by *psiTPTE22-HERV* (fold change = −1.34).

### *psiTPTE22-HERV* inhibits the PI3K/AKT/mTOR/EIF4E signaling pathway

We examined the impact of *psiTPTE22-HERV* on key proteins in the PI3K/AKT/mTOR signaling pathway, as well as on EIF4EBP1 and EIF4E at the protein level. Western blotting revealed that *psiTPTE22-HERV* down-regulated the levels of PI3K, p-AKT, mTOR, EIF4EBP1, p-EIF4EBP1 (at Thr70, driven by mTOR), and EIF4E, whereas *psiTPTE22-HERV* knockdown exhibited the opposite effects (Fig. 6D). Importantly, we observed significant inverse correlations between *psiTPTE22-HERV* expression and both EIF4E mRNA (*P* < 0.05) and protein levels (*P* < 0.0001) in primary gastric tumor tissues from the TCGA cohort (*n* = 331). Additionally, inverse correlations were observed with EIF4EBP1 protein (*P* < 0.0001) and pEIF4EBP1(Thr70) levels (*P* = 0.0003; Fig. 6E). Given the distinct roles of cytoplasmic and nuclear EIF4E,^23^ we assessed their levels in cytoplasmic and nuclear fractions. *psiTPTE22-HERV* down-regulated the cytoplasmic level of EIF4E, whereas *psiTPTE22-HERV* knockdown led to an increase in cytoplasmic EIF4E, with no changes observed in nuclear EIF4E (Fig. 6F). Further immunofluorescence staining confirmed the down-regulation of cytoplasmic EIF4E by *psiTPTE22-HERV* (Fig. 6G). These findings imply that the tumor-suppressive effect of *psiTPTE22-HERV* may be largely attributable to its negative regulation of cytoplasmic EIF4E via the PI3K/AKT/mTOR signaling pathway.

### Reduced *psiTPTE22-HERV* expression correlates with tumor progression and poor prognosis in gastric cancer

**Figure 7:**
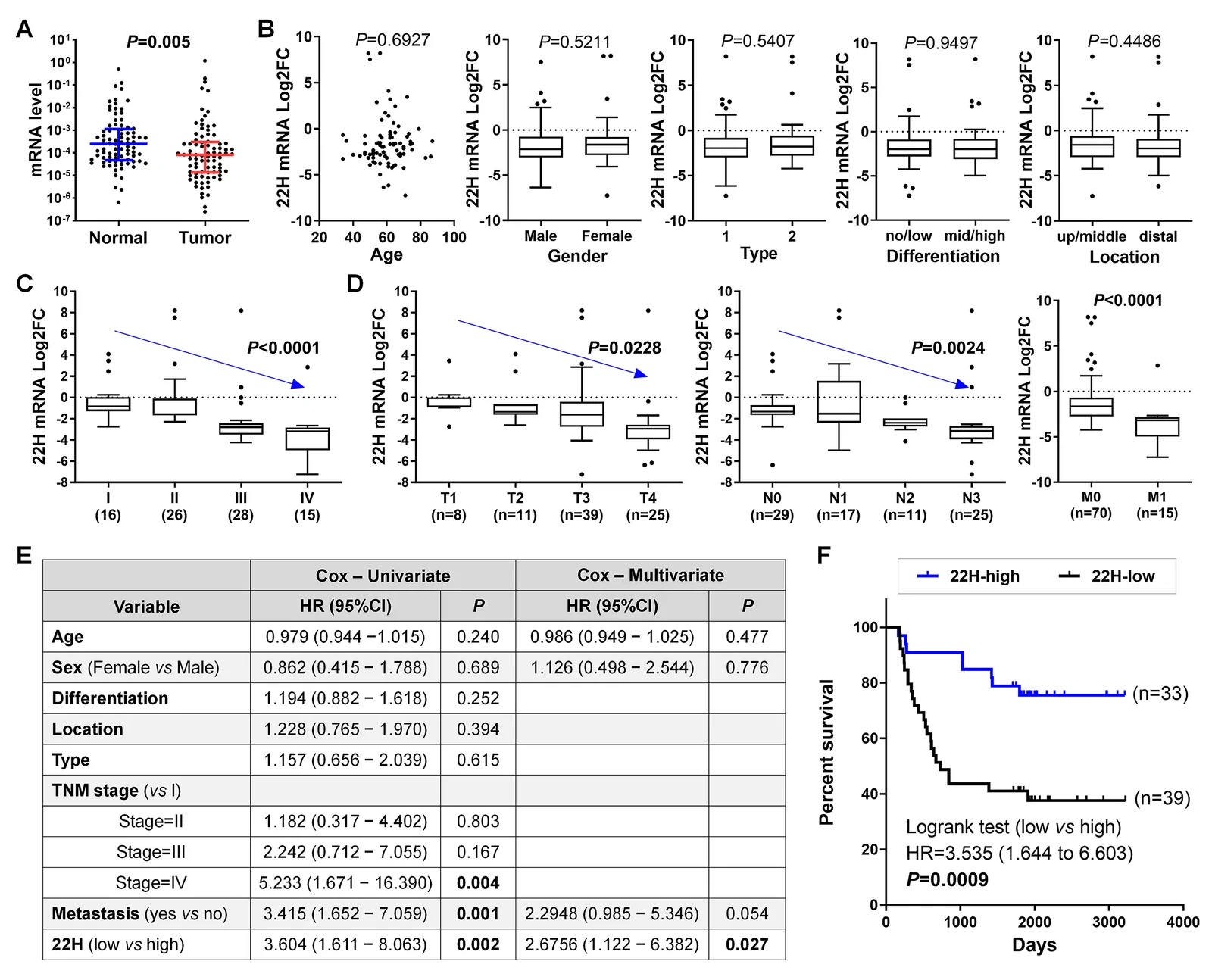
Clinical significance of *psiTPTE22-HERV* in gastric cancer. **(A)** Significant down-regulation of *psiTPTE22-HERV* in tumors versus paired non-tumor tissues (85 Chinese patients). **(B)** No association of *psiTPTE22-HERV* expression (log2 of fold change (FC) in tumor versus paired non-tumor) with age, sex, histological type (1: adenocarcinoma; 2: signet ring cell carcinoma), differentiation, or tumor location. **(C, D)** Progressive decrease of *psiTPTE22-HERV* expression with TNM stage advancement and individual T/N/M category progression. **(E)** Poor prognostic indicators revealed by univariate and multivariate Cox analysis. **(F)** The Kaplan–Meier survival curve showed shortened survival in patients with low *psiTPTE22-HERV* expression. The data were presented as median (interquartile range) (A) or box-and-whiskers (Tukey) (B–D) and compared using Mann–Whitney *U* test (A, B, D-right) or one-way ANOVA (C, D).

To investigate the clinical significance of *psiTPTE22-HERV*, we examined its expression in a cohort of 85 Chinese gastric cancer patients from a single center. *psiTPTE22-HERV* expression was significantly lower in tumors than in adjacent non-tumor tissues (Fig. 7A). The expression fold change in tumor versus adjacent non-tumor tissues was assessed, revealing no correlation with clinicopathological features such as age, sex, histological type, differentiation, or lesion location (all *P* > 0.05; Fig. 7B). However, we observed a significant linear decrease during tumor–node–metastasis (TNM) stage progression (*P* < 0.0001; Fig. 7C), with notable decreases associated with T, N, and M progression (all *P* < 0.05; Fig. 7D). These findings align with the *in vitro* and *in vivo* findings regarding the role of *psiTPTE22-HERV* in cell migration and metastasis to distant organs. Univariate Cox regression analysis indicated that *psiTPTE22-HERV* expression and TNM stage IV/metastasis, but not other clinicopathological features (age, sex, differentiation, tumor location, Lauren type), were significantly associated with shortened survival of gastric cancer patients. Furthermore, multivariate Cox regression analysis demonstrated that *psiTPTE22-HERV* expression was an independent predictor of poor survival (*P* < 0.05; Fig. 7E). Kaplan–Meier analysis showed that low levels of *psiTPTE22-HERV* expression were significantly associated with shortened survival in gastric cancer patients (Fig. 7F).

## Discussion

In this study, we explored the functional role, molecular mechanism, and clinical significance of *psiTPTE22-HERV* in gastric cancer. Consistently down-regulated across various tumor types, *psiTPTE22-HERV* plays an important role in suppressing the development and metastasis of gastric cancer. Its down-regulation in tumors is mediated by promoter methylation and serves as an independent risk factor for poor prognosis in gastric cancer patients. Notably, *psiTPTE22-HERV* exerted tumor-suppressive functions, at least partially, by down-regulating EIF4E via the PI3K/AKT/mTOR signaling pathway.

We demonstrated the tumor-suppressive function of *psiTPTE22-HERV* in gastric cancer both *in vitro* and *in vivo*. Using both gain- and loss-of-function assays, we found that *psiTPTE22-HERV* significantly suppressed gastric cancer cell growth by reducing cell viability and colony formation ability, inhibiting G1–S cell cycle transition, and inducing apoptosis. *In vivo* tumorigenesis assay further confirmed the tumor-suppressive role of *psiTPTE22-HERV* in suppressing the growth of subcutaneous xenografts. Supporting our clinical data, which showed that *psiTPTE22-HERV* down-regulation was correlated with tumor metastasis, we observed that *psiTPTE22-HERV* inhibited the migration and invasion of cancer cells *in vitro* and suppressed their metastasis to distant organs *in vivo*.

The molecular mechanism underlying the tumor-suppressive function of *psiTPTE22-HERV* may be associated with the suppression of protein synthesis with regard to the down-regulation of protein synthesis-related genes. Protein synthesis is essential for oncogenic cellular transformation.^24^ Specifically, we identified and validated two genes directly involved in protein synthesis initiation, EIF4EBP1 and EIF4E, which were down-regulated by *psiTPTE22-HERV*. The reverse regulation of EIF4EBP1 and EIF4E by *psiTPTE22-HERV* was demonstrated by the ectopic expression and knockdown of *psiTPTE22-HERV* in gastric cancer cells. Significant inverse correlations between *psiTPTE22-HERV* and both EIF4EBP1 and EIF4E were observed in primary gastric tumors. These results provide solid evidence supporting the effects of *psiTPTE22-HERV* on EIF4EBP1 and EIF4E, although these effects may not be direct but rather mediated through the suppression of the PI3K/AKT/mTOR pathway. The PI3K/AKT/mTOR pathway is known to be activated in gastric cancer, with accumulating evidence linking it to cell growth, metabolism, survival, metastasis, and resistance to chemotherapy.^25^ Increased EIF4E expression has been reported in gastric cancer, contributing to cell proliferation and disease progression.^26^ Moreover, overexpression of EIF4E is associated with tumor vascular invasion and lower survival rates in patients with gastric cancer.^27^ Phosphorylation of EIF4EBP1 can lead to the release of EIF4E, thereby relieving translational repression and enhancing oncogenic protein synthesis. However, our results showed that *psiTPTE22-HERV* decreased the levels of EIF4E, EIF4EBP1, and p-EIF4EBP1. The reduction in EIF4EBP1 levels may result from decreased EIF4E levels and contribute to lower p-EIF4EBP1 levels. Ultimately, the decrease in the oncogene EIF4E may significantly mediate the tumor-suppressive function of *psiTPTE22-HERV*.

Cytoplasmic and nuclear EIF4E play distinct roles: cytoplasmic EIF4E is essential for initiating protein synthesis, while nuclear EIF4E facilitates mRNA export.^23^ In our study, only cytoplasmic EIF4E levels were altered following *psiTPTE22-HERV* overexpression or knockdown, indicating that *psiTPTE22-HERV* primarily influences the regulation of protein synthesis. Hyperactive AKT can induce proteotoxic stress,^28^ characterized by the accumulation of misfolded or unfolded proteins. Additionally, mTOR is essential for the proteotoxic stress response.^29^ Notably, we observed that the expression of *psiTPTE22-HERV* led to the suppression of both AKT and mTOR. These findings suggest that *psiTPTE22-HERV* may help reduce cellular stress, ultimately resulting in decreased EIF4E expression.

While we have established that *psiTPTE22-HERV* is primarily regulated by promoter methylation at specific CpG sites, the specific transcription factors and binding motifs involved require further characterization. The molecular mechanism underlying its regulation of the PI3K/AKT/mTOR/EIF4E axis also requires further investigation. A major technical challenge in studying this gene is the lack of reliable antibodies. We made multiple attempts to generate antibodies using full-length proteins and synthetic peptides based on epitope prediction as immunogens, but the resulting antibodies were not satisfactory in terms of specificity and affinity. This limitation may hinder the characterization of its direct interacting molecules. Future efforts are needed to develop a specific and effective antibody for clinical applications, such as immunohistochemistry staining. Given its consistent down-regulation in various cancer types, the functional mechanisms and clinical implications of *psiTPTE22-HERV* in other cancers also merit exploration.

## Conclusions

In this study, we identified *psiTPTE22-HERV* as a novel tumor suppressor that was down-regulated by promoter methylation in gastric cancer. *psiTPTE22-HERV* exerts tumor-suppressive functions by down-regulating EIF4E via inhibition of the PI3K/AKT/mTOR pathway. Furthermore, down-regulation of *psiTPTE22-HERV* in primary tumors serves as an independent risk factor for poor prognosis in patients with gastric cancer.

## CRediT authorship contribution statement

**Fei Xu:** Writing – original draft, Methodology, Investigation, Formal analysis. **Mengwen Zhang:** Validation, Investigation. **Suzhan Zhang:** Resources, Project administration. **Yao Zeng:** Validation, Investigation. **Shu Zheng:** Supervision, Resources. **Jessie Qiaoyi Liang:** Writing – review & editing, Writing – original draft, Supervision, Funding acquisition, Data curation, Conceptualization.

## Ethics declaration

All animal experiments were conducted in accordance with institutional guidelines and were approved by the Animal Experimental Committee of Zhejiang University (#2016-239). This study adhered to the principles outlined in the Helsinki Declaration. This study was approved by the Clinical Research Ethics Committee of Zhejiang University (IRB# 2016-133). Written informed consent was obtained from all participants.

## Data availability

The RNA sequencing data generated in this study were deposited in the NCBI Sequence Read Archive (http://www.ncbi.nlm.nih.gov/sra) under accession number PRJNA803549.

## Funding

This project was supported by the Noncommunicable Chronic Diseases-National Science and Technology Major Project (China) (No. 2023ZD0501400) and the 10.13039/501100001809National Natural Science Foundation of China (No. 81773000).

## Conflict of interests

The authors declared no conflict of interests.
