# Supplementary material for: psiTPTE22-HERV functions as a tumor suppressor by inhibiting PI3K/AKT/mTOR/EIF4E signaling
Source: Genes Dis. 2025 Oct 31;13(6):101915. doi: 10.1016/j.gendis.2025.101915 (PMC13499165; doi:10.1016/j.gendis.2025.101915)
Supplement: Supplementary file 1 [file mmc1.docx]

**Table S1.** Characteristics of the cancer cell lines used in this study.

| **Cell line** | **Disease** | **Application** |
| --- | --- | --- |
| MKN45 | Gastric adenocarcinoma | RT-PCR, BGS, 5-Aza treatment, *in vitro*, and/or *in vivo* |
| SGC7901 | HeLa derivative; Human papillomavirus-related cervical adenocarcinoma |  |
| MKN1 | gastric adenosquamous carcinoma |  |
| AGS | Gastric adenocarcinoma | RT-PCR, BGS, 5-Aza treatment |
| NCI-N87 | Gastric tubular adenocarcinoma |  |
| SNU-719 | Gastric tubular adenocarcinoma |  |
| BGC823 | Gastric cancer cell lines | RT-PCR and/or BGS  (only cDNA and DNA from previous studies were used. No cell culture is involved in this study.) |
| KatoIII |  |  |
| MGC803 |  |  |
| MKN7 |  |  |
| MKN28 |  |  |
| MKN74 |  |  |
| SNU1 |  |  |
| SNU16 |  |  |
| Bcap37 | Breast adenocarcinoma (or HeLa derivative) | RT-PCR  (only cDNA was used in this study.) |
| MB231 | Breast adenocarcinoma |  |
| H1299 | Lung large cell carcinoma |  |
| H460 |  |  |
| A549 | Lung adenocarcinoma |  |
| SW480 | Colon adenocarcinoma |  |
| SW620 |  |  |
| Lovo |  |  |
| U87 | Glioblastoma |  |
| M059K |  |  |
| Hela | Human papillomavirus-related cervical adenocarcinoma |  |
| HOS | Osteosarcoma |  |
| U2OS |  |  |
| HepG2 | Hepatoblastoma |  |
| K562 | Chronic myeloid leukemia |  |
| KG-1a | Acute myeloid leukemia |  |
| THP-1 | Childhood acute monocytic leukemia |  |
| U937 | Adult acute monocytic leukemia |  |
| RPMI8226 | Multiple myeloma |  |
| KM3 |  |  |
| HEK293 | Transformed cell line of fetal kidney | RT-PCR (positive control) |

**Table S2.** Nucleotide sequences of the primers, probes and siRNA used in this study.

| **Primer Name** | **Sequence (5’-3’)** | **Size**  **/bp** | **Note** |
| --- | --- | --- | --- |
| psiTPTE22-HERV-F | ATGTCGTCGGAGGAGAACAAGT | 399 | RT-PCR |
| psiTPTE22-HERV-R | AGCCGAATAGATCAGATTTGAAATTG |  |  |
| Actin-F | GTCTTCCCCTCCATCGTG | 113 |  |
| Actin-R | AGGGTGAGGATGCCTCTCTT |  |  |
| psiTPTE22-HERV-qF | CCGGGATCCATGTGTCTTTC | 251 | RT-qPCR |
| psiTPTE22-HERV-qR | AGACCACCAAACAGGCTTCGT |  |  |
| psiTPTE22-HERV-probe | AGAAACTCAGCCCACCTGCACCCA |  |  |
| GAPDH-qF | TCGACAGTCAGCCGCATCT | 241 |  |
| GAPDH-qR | CTTGACGGTGCCATGGAATT |  |  |
| GAPDH-probe | CGTCGCCAGCCGAGCCACAT |  |  |
| psiTPTE22-HERV-BGS-F | TTTGGTTTAGTTGGATTATTGAGAAA | 230 | BGS |
| psiTPTE22-HERV-BGS-R | AATTTAAAACCAAAAAAAATTCCAC |  |  |
| siRNA-sense | AGAAACUCAGCCCACCUGCtt | NA | RNAi |
| siRNA-antisense | GCAGGUGGGCUGAGUUUCUtt |  |  |

**Table S3.** Antibodies used in this study.

| **Antibodies** | **Source** | **Identifier** |
| --- | --- | --- |
| P21 | Cell Signaling Technology, Danvers, MA | #2947 |
| P27 | Cell Signaling Technology | #2552 |
| CDK4 | Cell Signaling Technology | #12790 |
| Cyclin D3 | Cell Signaling Technology | #2936 |
| PCNA | Cell Signaling Technology | #2586 |
| Cleaved Caspase-9 | Cell Signaling Technology | #9501 |
| Cleaved Caspase-7 | Cell Signaling Technology | #9491 |
| Cleaved Caspase-3 | Cell Signaling Technology | #9661 |
| Cleaved PARP | Cell Signaling Technology | #9541 |
| N-Cadherin | Cell Signaling Technology | #13116 |
| E-Cadherin | Cell Signaling Technology | #3195 |
| β-catenin | Cell Signaling Technology | #8480 |
| Vimentin | Cell Signaling Technology | #5741 |
| Slug | Cell Signaling Technology | #9585 |
| Snail | Cell Signaling Technology | #3879 |
| TCF8/ZEB1 | Cell Signaling Technology | #3396 |
| 6×His-tag | abcam, Cambridge, UK | ab213204 |
| Ki67 | Abcam | ab15580 |
| PI3K | Cell Signaling Technology | #4292 |
| AKT | Cell Signaling Technology | #9272 |
| p-AKT (S308) | Cell Signaling Technology | #9275 |
| mTOR | HUABIO, Hangzhou, China | ET1608-5 |
| EIF4EBP1 | Cell Signaling Technology | #9452 |
| p-EIF4EBP1 (Thr70) | Cell Signaling Technology | #9455 |
| EIF4E | Abcam | ab33768 |
| GAPDH | Cell Signaling Technology | #5174 |
| Histone H3 | Cell Signaling Technology | #3638 |
| FITC AffiniPure® Goat Anti-Rabbit IgG (H+L) | Jackson ImmunoResearch, West Grove, PA | AB_2337972 |
| Anti-mouse IgG,HRP-linked | Cell Signaling Technology | #7076 |
| Anti-rabbit IgG,HRP-linked | Cell Signaling Technology | #7074 |

**Table S4.** Top differentially expressed genes identified by RNA sequencing for GO analysis.

| **Gene Symbol** | **Gene ID** | **22H_rc** | **ctrl_rc** | **FC*** | **P value** | **FDR*** |
| --- | --- | --- | --- | --- | --- | --- |
| NUPR1 | ENSG00000176046 | 34.775 | 123.566 | -3.553 | 2.53E-12 | 2.34E-09 |
| ASNS | ENSG00000070669 | 51.422 | 154.798 | -3.010 | 1.66E-12 | 1.69E-09 |
| LCN2 | ENSG00000148346 | 18.243 | 47.716 | -2.616 | 0.00043106 | 0.048152 |
| PSAT1 | ENSG00000135069 | 164.299 | 413.580 | -2.517 | 1.16E-23 | 3.75E-20 |
| TRIB3 | ENSG00000101255 | 45.379 | 113.279 | -2.496 | 1.91E-07 | 6.76E-05 |
| WARS | ENSG00000140105 | 70.007 | 170.167 | -2.431 | 4.68E-10 | 3.03E-07 |
| STC2 | ENSG00000113739 | 67.498 | 160.375 | -2.376 | 3.26E-09 | 1.71E-06 |
| CARS | ENSG00000110619 | 67.384 | 153.187 | -2.273 | 3.15E-08 | 1.42E-05 |
| GARS | ENSG00000106105 | 135.566 | 303.400 | -2.238 | 1.86E-14 | 2.26E-11 |
| EIF4EBP1 | ENSG00000187840 | 63.165 | 139.802 | -2.213 | 2.78E-07 | 9.16E-05 |
| MARS | ENSG00000166986 | 100.449 | 206.232 | -2.053 | 1.06E-08 | 5.27E-06 |
| FTH1 | ENSG00000167996 | 887.965 | 1733.022 | -1.952 | 1.79E-54 | 3.48E-50 |
| PHGDH | ENSG00000092621 | 342.507 | 665.794 | -1.944 | 9.92E-22 | 2.14E-18 |
| HSPB8 | ENSG00000152137 | 55.298 | 106.958 | -1.934 | 0.0001379 | 0.018876 |
| YARS | ENSG00000134684 | 177.069 | 339.713 | -1.919 | 1.85E-11 | 1.43E-08 |
| UPP1 | ENSG00000183696 | 58.947 | 112.535 | -1.909 | 0.00012331 | 0.017368 |
| FABP3 | ENSG00000121769 | 62.823 | 118.732 | -1.890 | 0.00010132 | 0.014481 |
| GDF15 | ENSG00000130513 | 133.172 | 248.619 | -1.867 | 3.28E-08 | 1.42E-05 |
| S100P | ENSG00000163993 | 144.802 | 267.086 | -1.844 | 1.86E-08 | 8.80E-06 |
| MTHFD2 | ENSG00000065911 | 97.941 | 176.487 | -1.802 | 1.03E-05 | 0.0023101 |
| SARS | ENSG00000031698 | 197.934 | 356.569 | -1.801 | 3.71E-10 | 2.49E-07 |
| SEC61G | ENSG00000132432 | 85.399 | 150.832 | -1.766 | 7.94E-05 | 0.012246 |
| ALPL | ENSG00000162551 | 90.188 | 159.012 | -1.763 | 5.34E-05 | 0.0088774 |
| CTD-2228K2.7 | ENSG00000225138 | 101.589 | 177.603 | -1.748 | 2.56E-05 | 0.0047916 |
| S100A11 | ENSG00000163191 | 165.667 | 288.031 | -1.739 | 1.09E-07 | 4.09E-05 |
| AARS | ENSG00000090861 | 339.543 | 587.465 | -1.730 | 5.42E-14 | 6.20E-11 |
| COX5B | ENSG00000135940 | 128.269 | 221.229 | -1.725 | 4.44E-06 | 0.0011066 |
| UBB | ENSG00000170315 | 124.393 | 211.314 | -1.699 | 1.27E-05 | 0.0027325 |
| ATF4 | ENSG00000128272 | 252.548 | 422.132 | -1.671 | 2.10E-09 | 1.14E-06 |
| PPP1R15A | ENSG00000087074 | 102.045 | 168.927 | -1.655 | 0.00019968 | 0.025703 |
| HAX1 | ENSG00000143575 | 102.273 | 168.431 | -1.647 | 0.00023611 | 0.029996 |
| LONP1 | ENSG00000196365 | 163.387 | 266.838 | -1.633 | 5.30E-06 | 0.0013048 |
| SHMT2 | ENSG00000182199 | 250.496 | 405.276 | -1.618 | 3.69E-08 | 1.49E-05 |
| FTL | ENSG00000087086 | 834.719 | 1329.976 | -1.593 | 4.31E-22 | 1.20E-18 |
| CLTB | ENSG00000175416 | 146.512 | 231.516 | -1.580 | 7.53E-05 | 0.011802 |
| HKDC1 | ENSG00000156510 | 139.329 | 220.113 | -1.580 | 0.00011423 | 0.016206 |
| MALAT1 | ENSG00000251562 | 1247.689 | 1969.619 | -1.579 | 9.29E-31 | 6.02E-27 |
| IARS | ENSG00000196305 | 236.357 | 371.070 | -1.570 | 7.76E-07 | 0.00022846 |
| SSR4 | ENSG00000180879 | 122.226 | 191.856 | -1.570 | 0.0003831 | 0.045129 |
| CGA | ENSG00000135346 | 259.047 | 406.144 | -1.568 | 2.55E-07 | 8.59E-05 |
| FAM129A | ENSG00000135842 | 161.448 | 252.213 | -1.562 | 5.60E-05 | 0.009152 |
| S100A6 | ENSG00000197956 | 394.271 | 615.475 | -1.561 | 3.29E-10 | 2.29E-07 |
| EIF1 | ENSG00000173812 | 472.259 | 737.058 | -1.561 | 6.24E-12 | 5.27E-09 |
| GHITM | ENSG00000165678 | 268.852 | 419.157 | -1.559 | 2.33E-07 | 8.09E-05 |
| TCEB2 | ENSG00000103363 | 168.175 | 261.757 | -1.556 | 4.69E-05 | 0.0081366 |
| EIF2S2 | ENSG00000125977 | 144.916 | 225.443 | -1.556 | 0.00016108 | 0.021445 |
| JTB | ENSG00000143543 | 151.985 | 234.118 | -1.540 | 0.00017065 | 0.022347 |
| RPS29 | ENSG00000213741 | 249.697 | 379.621 | -1.520 | 3.49E-06 | 0.00090434 |
| 22H | ENSG00000100181 | 18.927 | 2.727 | 6.941 | 0.00017131 | 0.022347 |
| HSPG2 | ENSG00000142798 | 510.455 | 262.128 | 1.947 | 6.22E-22 | 1.51E-18 |
| PTGS1 | ENSG00000095303 | 117.666 | 67.422 | 1.745 | 5.87E-05 | 0.0094372 |
| NOTCH1 | ENSG00000148400 | 149.476 | 89.979 | 1.661 | 2.56E-05 | 0.0047916 |
| EPPK1 | ENSG00000227184 | 342.507 | 210.942 | 1.624 | 8.08E-10 | 4.91E-07 |
| ADAMTSL4 | ENSG00000143382 | 132.944 | 84.526 | 1.573 | 0.00028794 | 0.035648 |
| FOXE1 | ENSG00000178919 | 173.762 | 110.800 | 1.568 | 3.72E-05 | 0.0067599 |
| LAMA5 | ENSG00000130702 | 1329.782 | 858.765 | 1.548 | 6.61E-29 | 3.21E-25 |
| LTBP4 | ENSG00000090006 | 174.788 | 114.395 | 1.528 | 8.00E-05 | 0.012246 |
| LRP1 | ENSG00000123384 | 219.255 | 145.255 | 1.509 | 1.59E-05 | 0.0032482 |
| GOLGA8A | ENSG00000175265 | 153.923 | 102.125 | 1.507 | 0.0003111 | 0.03803 |
| FASN | ENSG00000169710 | 434.177 | 289.147 | 1.502 | 1.83E-09 | 1.04E-06 |

*Genes with |fold-change| >=1.5 and FDR<0.05.

**Table S5.** GO biological processes identified by Over-Representation Analysis.

| **Gene Set** | **Description** | **Overlap** | **Gene Set Size** | **Expect value** | **Enrichment Ratio** | **P Value** | **FDR** | **Genes** |
| --- | --- | --- | --- | --- | --- | --- | --- | --- |
| **GO:0006520** | **cellular amino acid metabolic process** | **13** | 318 | 1.1597 | 11.21 | 8.14E-11 | 6.92E-08 | ASNS, PSAT1, WARS, CARS, GARS, MARS, PHGDH, YARS, SARS, AARS, ATF4, SHMT2, IARS |
| **GO:0006399** | **tRNA metabolic process** | **8** | 183 | 0.66739 | 11.987 | 2.99E-07 | 0.000127 | WARS, CARS, GARS, MARS, YARS, SARS, AARS, IARS |
| **GO:0006413** | **translational initiation** | **6** | 192 | 0.70021 | 8.5689 | 6.77E-05 | 0.019175 | **EIF4EBP1**, ATF4, PPP1R15A, EIF1, EIF2S2, RPS29 |
| **GO:0010608** | **posttranscriptional regulation of gene expression** | **8** | 486 | 1.7724 | 4.5136 | 0.000351 | 0.043159 | **EIF4EBP1**, AARS, UBB, ATF4, PPP1R15A, SHMT2, FAM129A, EIF1 |
| **GO:1901652** | **response to peptide** | **8** | 487 | 1.7761 | 4.5044 | 0.000355 | 0.043159 | **EIF4EBP1**, TRIB3, STC2, MARS, FABP3, GDF15, NOTCH1, LRP1 |
| **GO:0034248** | **regulation of cellular amide metabolic process** | **7** | 385 | 1.4041 | 4.9855 | 0.00047 | 0.044393 | **EIF4EBP1**, AARS, ATF4, PPP1R15A, SHMT2, FAM129A, EIF1 |
| **GO:0035966** | **Response to topologically incorrect protein** | **5** | 188 | 0.68562 | 7.2927 | 0.000598 | 0.046182 | ASNS, STC2, HSPB8, ATF4, PPP1R15A |
| GO:0034976 | response to endoplasmic reticulum stress | 6 | 268 | 0.97738 | 6.1389 | 0.000414 | 0.044031 | ASNS, TRIB3, STC2, ATF4, PPP1R15A, FAM129A |
| GO:0016053 | organic acid biosynthetic process | 8 | 401 | 1.4624 | 5.4704 | 9.43E-05 | 0.020041 | ASNS, PSAT1, TRIB3, PHGDH, SHMT2, HKDC1, PTGS1, FASN |
| GO:0031667 | response to nutrient levels | 8 | 483 | 1.7615 | 4.5417 | 0.000336 | 0.043159 | ASNS, LCN2, STC2, MARS, UPP1, GDF15, ALPL, ATF4 |

**Table S6.** Differentially expressed genes for pathway enrichment analysis.

| **Gene Symbol** | **Gene ID** | **22H_rc** | **ctrl_rc** | **FC*** | **P value** |
| --- | --- | --- | --- | --- | --- |
| ADM2 | ENSG00000128165 | 6.499 | 24.416 | -3.757 | 0.0013319 |
| NUPR1 | ENSG00000176046 | 34.775 | 123.566 | -3.553 | 2.53E-12 |
| ASNS | ENSG00000070669 | 51.422 | 154.798 | -3.010 | 1.66E-12 |
| DDIT3 | ENSG00000175197 | 5.929 | 17.351 | -2.927 | 0.020503 |
| RENBP | ENSG00000102032 | 11.288 | 31.852 | -2.822 | 0.0022446 |
| HMOX1 | ENSG00000100292 | 9.349 | 25.035 | -2.678 | 0.0093247 |
| LCN2 | ENSG00000148346 | 18.243 | 47.716 | -2.616 | 0.0004311 |
| MIR22HG | ENSG00000186594 | 7.867 | 20.326 | -2.584 | 0.022985 |
| SPINK1 | ENSG00000164266 | 7.183 | 18.343 | -2.554 | 0.032496 |
| PSAT1 | ENSG00000135069 | 164.299 | 413.580 | -2.517 | 1.16E-23 |
| TRIB3 | ENSG00000101255 | 45.379 | 113.279 | -2.496 | 1.91E-07 |
| SESN2 | ENSG00000130766 | 20.409 | 50.814 | -2.490 | 0.0005014 |
| WARS | ENSG00000140105 | 70.007 | 170.167 | -2.431 | 4.68E-10 |
| COX6A1 | ENSG00000111775 | 8.665 | 20.945 | -2.417 | 0.029707 |
| PSPH | ENSG00000146733 | 21.321 | 50.938 | -2.389 | 0.0008001 |
| STC2 | ENSG00000113739 | 67.498 | 160.375 | -2.376 | 3.26E-09 |
| CARS | ENSG00000110619 | 67.384 | 153.187 | -2.273 | 3.15E-08 |
| GRB10 | ENSG00000106070 | 21.435 | 48.584 | -2.267 | 0.0018952 |
| GARS | ENSG00000106105 | 135.566 | 303.400 | -2.238 | 1.86E-14 |
| RP11-48O20.4 | ENSG00000224259 | 24.970 | 55.524 | -2.224 | 0.0011374 |
| EIF4EBP1 | ENSG00000187840 | 63.165 | 139.802 | -2.213 | 2.78E-07 |
| CBS | ENSG00000160200 | 27.022 | 57.507 | -2.128 | 0.001614 |
| COX8A | ENSG00000176340 | 12.200 | 25.903 | -2.123 | 0.034803 |
| ALDH1L2 | ENSG00000136010 | 29.416 | 62.093 | -2.111 | 0.0011721 |
| NEU1 | ENSG00000204386 | 14.708 | 30.365 | -2.064 | 0.0271 |
| MARS | ENSG00000166986 | 100.449 | 206.232 | -2.053 | 1.06E-08 |
| GADD45A | ENSG00000116717 | 40.134 | 80.312 | -2.001 | 0.0005477 |
| FTH1 | ENSG00000167996 | 887.965 | 1733.022 | -1.952 | 1.79E-54 |
| PHGDH | ENSG00000092621 | 342.507 | 665.794 | -1.944 | 9.92E-22 |
| HSPB8 | ENSG00000152137 | 55.298 | 106.958 | -1.934 | 0.0001379 |
| SLIRP | ENSG00000119705 | 18.243 | 35.198 | -1.929 | 0.029313 |
| YARS | ENSG00000134684 | 177.069 | 339.713 | -1.919 | 1.85E-11 |
| UPP1 | ENSG00000183696 | 58.947 | 112.535 | -1.909 | 0.0001233 |
| PCK2 | ENSG00000100889 | 38.766 | 73.867 | -1.905 | 0.0019208 |
| FABP3 | ENSG00000121769 | 62.823 | 118.732 | -1.890 | 0.0001013 |
| GDF15 | ENSG00000130513 | 133.172 | 248.619 | -1.867 | 3.28E-08 |
| NME1 | ENSG00000239672 | 21.435 | 39.784 | -1.856 | 0.02844 |
| S100P | ENSG00000163993 | 144.802 | 267.086 | -1.844 | 1.86E-08 |
| NDUFB1 | ENSG00000183648 | 26.110 | 48.088 | -1.842 | 0.017257 |
| TNNC1 | ENSG00000114854 | 45.493 | 83.534 | -1.836 | 0.0017837 |
| ISG15 | ENSG00000187608 | 44.353 | 81.055 | -1.828 | 0.0022503 |
| NNMT | ENSG00000166741 | 39.792 | 72.380 | -1.819 | 0.0041574 |
| MTHFD2 | ENSG00000065911 | 97.941 | 176.487 | -1.802 | 1.03E-05 |
| SARS | ENSG00000031698 | 197.934 | 356.569 | -1.801 | 3.71E-10 |
| CDKN1A | ENSG00000124762 | 23.031 | 41.395 | -1.797 | 0.033431 |
| EIF1B | ENSG00000114784 | 36.371 | 65.191 | -1.792 | 0.0078767 |
| UQCRQ | ENSG00000164405 | 43.441 | 77.833 | -1.792 | 0.0037098 |
| IDH1 | ENSG00000138413 | 52.790 | 93.325 | -1.768 | 0.0018784 |
| SEC61G | ENSG00000132432 | 85.399 | 150.832 | -1.766 | 7.94E-05 |
| ALPL | ENSG00000162551 | 90.188 | 159.012 | -1.763 | 5.34E-05 |
| SEL1L3 | ENSG00000091490 | 27.820 | 48.955 | -1.760 | 0.025461 |
| ATP5L | ENSG00000167283 | 31.355 | 54.904 | -1.751 | 0.01894 |
| COA6 | ENSG00000168275 | 50.510 | 88.368 | -1.750 | 0.0029516 |
| ARL2BP | ENSG00000102931 | 24.172 | 42.263 | -1.748 | 0.040009 |
| CTD-2228K2.7 | ENSG00000225138 | 101.589 | 177.603 | -1.748 | 2.56E-05 |
| TXNDC17 | ENSG00000129235 | 28.846 | 50.319 | -1.744 | 0.025616 |
| S100A11 | ENSG00000163191 | 165.667 | 288.031 | -1.739 | 1.09E-07 |
| GOT1 | ENSG00000120053 | 32.951 | 57.011 | -1.730 | 0.019148 |
| AARS | ENSG00000090861 | 339.543 | 587.465 | -1.730 | 5.42E-14 |
| LAMTOR5 | ENSG00000134248 | 36.257 | 62.713 | -1.730 | 0.014064 |
| COX5B | ENSG00000135940 | 128.269 | 221.229 | -1.725 | 4.44E-06 |
| LARP6 | ENSG00000166173 | 26.908 | 46.229 | -1.718 | 0.037197 |
| RPL26L1 | ENSG00000037241 | 71.945 | 123.566 | -1.718 | 0.0006622 |
| ETV5 | ENSG00000244405 | 31.127 | 53.417 | -1.716 | 0.025394 |
| UBB | ENSG00000170315 | 124.393 | 211.314 | -1.699 | 1.27E-05 |
| COX7B | ENSG00000131174 | 72.743 | 123.566 | -1.699 | 0.0008432 |
| TNFRSF14 | ENSG00000157873 | 32.381 | 54.780 | -1.692 | 0.027372 |
| C14orf2 | ENSG00000156411 | 43.783 | 73.991 | -1.690 | 0.010497 |
| SRA1 | ENSG00000213523 | 31.811 | 53.417 | -1.679 | 0.031649 |
| HERPUD1 | ENSG00000051108 | 69.208 | 116.130 | -1.678 | 0.001556 |
| ATF4 | ENSG00000128272 | 252.548 | 422.132 | -1.671 | 2.10E-09 |
| COX14 | ENSG00000178449 | 56.210 | 93.697 | -1.667 | 0.005003 |
| PPP1R15A | ENSG00000087074 | 102.045 | 168.927 | -1.655 | 0.0001997 |
| NDUFAB1 | ENSG00000004779 | 43.098 | 71.264 | -1.654 | 0.015936 |
| HAX1 | ENSG00000143575 | 102.273 | 168.431 | -1.647 | 0.0002361 |
| NDUFA1 | ENSG00000125356 | 76.391 | 125.177 | -1.639 | 0.0016976 |
| ALDH2 | ENSG00000111275 | 47.887 | 78.329 | -1.636 | 0.013365 |
| LONP1 | ENSG00000196365 | 163.387 | 266.838 | -1.633 | 5.30E-06 |
| SLC35F6 | ENSG00000213699 | 40.134 | 65.191 | -1.624 | 0.02604 |
| C9orf91 | ENSG00000157693 | 35.003 | 56.640 | -1.618 | 0.039552 |
| SHMT2 | ENSG00000182199 | 250.496 | 405.276 | -1.618 | 3.69E-08 |
| GPT2 | ENSG00000166123 | 36.942 | 59.738 | -1.617 | 0.034755 |
| PRR13 | ENSG00000205352 | 53.816 | 87.004 | -1.617 | 0.010878 |
| MAP1LC3B | ENSG00000140941 | 79.356 | 128.276 | -1.616 | 0.0019929 |
| F3 | ENSG00000117525 | 72.401 | 116.873 | -1.614 | 0.0032565 |
| TCEA1 | ENSG00000187735 | 89.161 | 143.520 | -1.610 | 0.0011886 |
| COX6C | ENSG00000164919 | 52.220 | 84.030 | -1.609 | 0.013186 |
| CHCHD1 | ENSG00000172586 | 52.676 | 84.402 | -1.602 | 0.013812 |
| RRAGC | ENSG00000116954 | 54.614 | 87.500 | -1.602 | 0.012194 |
| GNG5 | ENSG00000174021 | 45.151 | 72.008 | -1.595 | 0.024327 |
| FTL | ENSG00000087086 | 834.719 | 1329.976 | -1.593 | 4.31E-22 |
| SLC31A1 | ENSG00000136868 | 74.111 | 117.369 | -1.584 | 0.0046224 |
| ROMO1 | ENSG00000125995 | 38.994 | 61.721 | -1.583 | 0.040226 |
| CIB1 | ENSG00000185043 | 65.674 | 103.860 | -1.581 | 0.0079054 |
| CLTB | ENSG00000175416 | 146.512 | 231.516 | -1.580 | 7.53E-05 |
| HKDC1 | ENSG00000156510 | 139.329 | 220.113 | -1.580 | 0.0001142 |
| MALAT1 | ENSG00000251562 | 1247.689 | 1969.619 | -1.579 | 9.29E-31 |
| MRPS15 | ENSG00000116898 | 70.919 | 111.792 | -1.576 | 0.0062136 |
| COX7A2 | ENSG00000112695 | 96.687 | 152.320 | -1.575 | 0.0014234 |
| MMADHC | ENSG00000168288 | 52.334 | 82.419 | -1.575 | 0.019047 |
| IARS | ENSG00000196305 | 236.357 | 371.070 | -1.570 | 7.76E-07 |
| CEACAM7 | ENSG00000007306 | 37.740 | 59.242 | -1.570 | 0.048424 |
| SSR4 | ENSG00000180879 | 122.226 | 191.856 | -1.570 | 0.0003831 |
| CGA | ENSG00000135346 | 259.047 | 406.144 | -1.568 | 2.55E-07 |
| FAM129A | ENSG00000135842 | 161.448 | 252.213 | -1.562 | 5.60E-05 |
| S100A6 | ENSG00000197956 | 394.271 | 615.475 | -1.561 | 3.29E-10 |
| EIF1 | ENSG00000173812 | 472.259 | 737.058 | -1.561 | 6.24E-12 |
| SRPRB | ENSG00000144867 | 73.313 | 114.395 | -1.560 | 0.0068087 |
| GHITM | ENSG00000165678 | 268.852 | 419.157 | -1.559 | 2.33E-07 |
| TCEB2 | ENSG00000103363 | 168.175 | 261.757 | -1.556 | 4.69E-05 |
| EIF2S2 | ENSG00000125977 | 144.916 | 225.443 | -1.556 | 0.0001611 |
| FOSL1 | ENSG00000175592 | 45.493 | 70.149 | -1.542 | 0.039177 |
| JTB | ENSG00000143543 | 151.985 | 234.118 | -1.540 | 0.0001707 |
| GTPBP2 | ENSG00000172432 | 110.141 | 168.307 | -1.528 | 0.0017642 |
| NDUFA4 | ENSG00000189043 | 116.297 | 177.355 | -1.525 | 0.0013999 |
| SEC61B | ENSG00000106803 | 75.479 | 115.014 | -1.524 | 0.010241 |
| SNRPC | ENSG00000124562 | 76.505 | 116.378 | -1.521 | 0.010109 |
| RPS29 | ENSG00000213741 | 249.697 | 379.621 | -1.520 | 3.49E-06 |
| MTMR14 | ENSG00000163719 | 47.773 | 72.380 | -1.515 | 0.044576 |
| EMC7 | ENSG00000134153 | 76.277 | 115.262 | -1.511 | 0.011787 |
| PEPD | ENSG00000124299 | 62.595 | 94.565 | -1.511 | 0.022621 |
| EGR1 | ENSG00000120738 | 68.524 | 103.364 | -1.508 | 0.017571 |
| ASAH1 | ENSG00000104763 | 86.539 | 130.506 | -1.508 | 0.0076627 |
| CTSD | ENSG00000117984 | 136.022 | 205.117 | -1.508 | 0.0008301 |
| SLC1A5 | ENSG00000105281 | 299.523 | 448.779 | -1.498 | 1.14E-06 |
| HSPA9 | ENSG00000113013 | 488.563 | 730.861 | -1.496 | 6.21E-10 |
| ARL8B | ENSG00000134108 | 54.272 | 81.179 | -1.496 | 0.039375 |
| HN1 | ENSG00000189159 | 88.021 | 131.622 | -1.495 | 0.0087552 |
| RP11-492E3.1 | ENSG00000204054 | 119.604 | 178.842 | -1.495 | 0.0022469 |
| ERP29 | ENSG00000089248 | 143.206 | 213.917 | -1.494 | 0.0008587 |
| HSPA5 | ENSG00000044574 | 657.537 | 980.472 | -1.491 | 1.20E-12 |
| NHP2L1 | ENSG00000100138 | 107.518 | 160.252 | -1.490 | 0.0041248 |
| IFRD1 | ENSG00000006652 | 80.268 | 119.476 | -1.488 | 0.01358 |
| LGALS1 | ENSG00000100097 | 153.467 | 228.417 | -1.488 | 0.000644 |
| HLA-A | ENSG00000206503 | 213.896 | 318.272 | -1.488 | 5.69E-05 |
| TARS | ENSG00000113407 | 444.781 | 661.456 | -1.487 | 6.81E-09 |
| PRDX5 | ENSG00000126432 | 145.372 | 216.147 | -1.487 | 0.0009307 |
| RTFDC1 | ENSG00000022277 | 62.709 | 93.201 | -1.486 | 0.029893 |
| BLVRB | ENSG00000090013 | 59.403 | 88.244 | -1.486 | 0.034835 |
| SDHB | ENSG00000117118 | 58.263 | 86.509 | -1.485 | 0.036905 |
| C19orf10 | ENSG00000074842 | 106.606 | 158.269 | -1.485 | 0.0047761 |
| NELFE | ENSG00000204356 | 73.883 | 109.685 | -1.485 | 0.01883 |
| NOP10 | ENSG00000182117 | 66.586 | 98.654 | -1.482 | 0.026691 |
| GFPT1 | ENSG00000198380 | 200.442 | 296.211 | -1.478 | 0.0001367 |
| XPOT | ENSG00000184575 | 249.469 | 368.343 | -1.477 | 2.20E-05 |
| TTC1 | ENSG00000113312 | 56.667 | 83.534 | -1.474 | 0.044217 |
| ODC1 | ENSG00000115758 | 249.812 | 367.971 | -1.473 | 2.50E-05 |
| NDUFB4 | ENSG00000065518 | 95.204 | 140.174 | -1.472 | 0.0093772 |
| PAGE1 | ENSG00000068985 | 60.543 | 89.111 | -1.472 | 0.038492 |
| SRP14 | ENSG00000140319 | 135.566 | 199.168 | -1.469 | 0.0020777 |
| ATP6V0E1 | ENSG00000113732 | 219.027 | 321.371 | -1.467 | 9.71E-05 |
| COX5A | ENSG00000178741 | 96.687 | 141.785 | -1.466 | 0.0097452 |
| PSMB1 | ENSG00000008018 | 86.425 | 126.664 | -1.466 | 0.014719 |
| CKS2 | ENSG00000123975 | 68.638 | 100.390 | -1.463 | 0.03082 |
| SIVA1 | ENSG00000184990 | 59.859 | 87.500 | -1.462 | 0.044125 |
| CSTB | ENSG00000160213 | 94.862 | 138.562 | -1.461 | 0.011476 |
| CSF2RA | ENSG00000198223 | 69.436 | 101.381 | -1.460 | 0.030792 |
| MT2A | ENSG00000125148 | 111.623 | 162.482 | -1.456 | 0.0066989 |
| AVPI1 | ENSG00000119986 | 236.243 | 343.184 | -1.453 | 8.91E-05 |
| PTGES | ENSG00000148344 | 65.788 | 95.556 | -1.452 | 0.038756 |
| ANXA2 | ENSG00000182718 | 764.941 | 1110.234 | -1.451 | 2.02E-12 |
| TXN | ENSG00000136810 | 377.625 | 547.929 | -1.451 | 7.99E-07 |
| CTSL1 | ENSG00000135047 | 117.894 | 170.910 | -1.450 | 0.0059728 |
| EIF3K | ENSG00000178982 | 81.978 | 118.732 | -1.448 | 0.02229 |
| ATP5F1 | ENSG00000116459 | 113.219 | 163.970 | -1.448 | 0.0072464 |
| GCLM | ENSG00000023909 | 80.496 | 116.501 | -1.447 | 0.023871 |
| ATP6V0B | ENSG00000117410 | 77.760 | 112.535 | -1.447 | 0.026412 |
| ID3 | ENSG00000117318 | 165.439 | 239.200 | -1.446 | 0.0012456 |
| HYOU1 | ENSG00000149428 | 141.609 | 204.497 | -1.444 | 0.0029343 |
| PSMC1 | ENSG00000100764 | 84.943 | 122.451 | -1.442 | 0.022013 |
| PTTG1IP | ENSG00000183255 | 189.610 | 273.283 | -1.441 | 0.0006272 |
| GCLC | ENSG00000001084 | 66.472 | 95.804 | -1.441 | 0.042921 |
| GPNMB | ENSG00000136235 | 109.913 | 158.269 | -1.440 | 0.0094508 |
| CHCHD2 | ENSG00000106153 | 269.993 | 388.173 | -1.438 | 5.19E-05 |
| VAPA | ENSG00000101558 | 89.275 | 128.276 | -1.437 | 0.020235 |
| TPRA1 | ENSG00000163870 | 275.351 | 395.361 | -1.436 | 4.73E-05 |
| RPL36 | ENSG00000130255 | 210.818 | 302.656 | -1.436 | 0.0003736 |
| OAZ1 | ENSG00000104904 | 192.917 | 276.753 | -1.435 | 0.0006861 |
| KRT80 | ENSG00000167767 | 378.993 | 543.343 | -1.434 | 2.05E-06 |
| ATP5E | ENSG00000124172 | 125.647 | 179.834 | -1.431 | 0.0065636 |
| SLC6A9 | ENSG00000196517 | 72.971 | 104.356 | -1.430 | 0.038864 |
| COX6B1 | ENSG00000126267 | 105.694 | 151.080 | -1.429 | 0.013067 |
| ID1 | ENSG00000125968 | 153.011 | 217.511 | -1.422 | 0.0033957 |
| SNRPG | ENSG00000143977 | 91.442 | 129.887 | -1.420 | 0.023923 |
| LSM3 | ENSG00000170860 | 74.795 | 106.215 | -1.420 | 0.041287 |
| PDIA3 | ENSG00000167004 | 226.666 | 321.371 | -1.418 | 0.0004111 |
| SBDS | ENSG00000126524 | 100.107 | 141.909 | -1.418 | 0.018967 |
| VAT1 | ENSG00000108828 | 303.628 | 429.940 | -1.416 | 4.69E-05 |
| CEP72 | ENSG00000112877 | 87.223 | 123.194 | -1.412 | 0.030641 |
| SEC61A1 | ENSG00000058262 | 513.533 | 725.284 | -1.412 | 1.56E-07 |
| TBC1D2 | ENSG00000095383 | 108.316 | 152.691 | -1.410 | 0.016741 |
| TSPO | ENSG00000100300 | 105.808 | 148.849 | -1.407 | 0.018926 |
| NQO1 | ENSG00000181019 | 170.228 | 238.952 | -1.404 | 0.0031421 |
| CLIC4 | ENSG00000169504 | 377.853 | 530.330 | -1.404 | 1.09E-05 |
| SKP1 | ENSG00000113558 | 193.601 | 271.672 | -1.403 | 0.0016555 |
| CD63 | ENSG00000135404 | 535.196 | 750.071 | -1.401 | 1.91E-07 |
| EDF1 | ENSG00000107223 | 288.805 | 404.037 | -1.399 | 0.0001447 |
| NDUFS5 | ENSG00000168653 | 168.289 | 235.358 | -1.399 | 0.0037682 |
| POLR2L | ENSG00000177700 | 104.554 | 145.999 | -1.396 | 0.023181 |
| PGK1 | ENSG00000102144 | 622.762 | 868.184 | -1.394 | 3.61E-08 |
| SNRPF | ENSG00000139343 | 89.047 | 123.938 | -1.392 | 0.038474 |
| PSAP | ENSG00000197746 | 1703.302 | 2369.194 | -1.391 | 1.57E-19 |
| H1F0 | ENSG00000189060 | 137.505 | 191.236 | -1.391 | 0.010326 |
| MT-ND3 | ENSG00000198840 | 535.652 | 743.503 | -1.388 | 5.01E-07 |
| SLC39A1 | ENSG00000143570 | 327.343 | 454.356 | -1.388 | 8.56E-05 |
| SQSTM1 | ENSG00000161011 | 614.324 | 850.709 | -1.385 | 9.56E-08 |
| CUTA | ENSG00000112514 | 143.662 | 198.796 | -1.384 | 0.010112 |
| MYL6 | ENSG00000092841 | 314.345 | 434.774 | -1.383 | 0.000146 |
| PFDN5 | ENSG00000123349 | 191.321 | 264.607 | -1.383 | 0.0030543 |
| EMC4 | ENSG00000128463 | 92.924 | 128.152 | -1.379 | 0.041182 |
| SDHC | ENSG00000143252 | 91.328 | 125.673 | -1.376 | 0.044769 |
| PSMC5 | ENSG00000087191 | 95.660 | 131.622 | -1.376 | 0.040064 |
| PDCD5 | ENSG00000105185 | 108.544 | 149.345 | -1.376 | 0.028761 |
| MYL12B | ENSG00000118680 | 292.568 | 402.426 | -1.375 | 0.0003352 |
| ATPIF1 | ENSG00000130770 | 121.542 | 166.696 | -1.372 | 0.022299 |
| RNF187 | ENSG00000168159 | 207.283 | 284.065 | -1.370 | 0.0029282 |
| PSMA7 | ENSG00000101182 | 291.428 | 399.327 | -1.370 | 0.0004217 |
| EI24 | ENSG00000149547 | 113.789 | 155.914 | -1.370 | 0.027608 |
| CDK4 | ENSG00000135446 | 122.682 | 167.936 | -1.369 | 0.022698 |
| BNIP3L | ENSG00000104765 | 114.017 | 156.038 | -1.369 | 0.0282 |
| ETFA | ENSG00000140374 | 142.065 | 194.210 | -1.367 | 0.014728 |
| FAU | ENSG00000149806 | 153.923 | 210.322 | -1.366 | 0.011276 |
| ARF4 | ENSG00000168374 | 179.349 | 245.025 | -1.366 | 0.0062644 |
| EIF5A | ENSG00000132507 | 459.945 | 627.497 | -1.364 | 1.34E-05 |
| PSMB7 | ENSG00000136930 | 303.058 | 412.589 | -1.361 | 0.0004582 |
| RBM3 | ENSG00000102317 | 471.119 | 640.882 | -1.360 | 1.33E-05 |
| NDUFS6 | ENSG00000145494 | 206.941 | 281.339 | -1.360 | 0.0039898 |
| LCP1 | ENSG00000136167 | 265.660 | 360.411 | -1.357 | 0.0012196 |
| TMEM14C | ENSG00000111843 | 176.841 | 239.572 | -1.355 | 0.0087096 |
| SSR2 | ENSG00000163479 | 174.788 | 236.721 | -1.354 | 0.009193 |
| RPL38 | ENSG00000172809 | 267.712 | 362.394 | -1.354 | 0.0012938 |
| ATP5G3 | ENSG00000154518 | 100.563 | 135.960 | -1.352 | 0.049813 |
| RAB1A | ENSG00000138069 | 113.105 | 152.815 | -1.351 | 0.038023 |
| ENSA | ENSG00000143420 | 231.797 | 312.199 | -1.347 | 0.0033734 |
| C1orf43 | ENSG00000143612 | 256.310 | 345.167 | -1.347 | 0.0020645 |
| UQCRH | ENSG00000173660 | 281.622 | 378.754 | -1.345 | 0.0013206 |
| FAM50A | ENSG00000071859 | 111.737 | 150.213 | -1.344 | 0.043474 |
| TNFRSF12A | ENSG00000006327 | 135.452 | 182.065 | -1.344 | 0.026311 |
| SSR3 | ENSG00000114850 | 249.583 | 335.376 | -1.344 | 0.0025936 |
| RPL37 | ENSG00000145592 | 2429.591 | 3260.307 | -1.342 | 8.30E-21 |
| SPCS2 | ENSG00000118363 | 135.566 | 181.569 | -1.339 | 0.028602 |
| TXNRD1 | ENSG00000198431 | 864.136 | 1157.331 | -1.339 | 3.24E-08 |
| ARHGEF2 | ENSG00000116584 | 117.894 | 157.773 | -1.338 | 0.041928 |
| PEA15 | ENSG00000162734 | 134.312 | 179.710 | -1.338 | 0.030049 |
| MGST3 | ENSG00000143198 | 139.329 | 186.402 | -1.338 | 0.027203 |
| RPN2 | ENSG00000118705 | 210.704 | 281.834 | -1.338 | 0.0066529 |
| ANXA5 | ENSG00000164111 | 560.622 | 749.452 | -1.337 | 1.00E-05 |
| SLC3A2 | ENSG00000168003 | 670.193 | 894.335 | -1.334 | 1.65E-06 |
| SLC7A11 | ENSG00000151012 | 197.934 | 264.111 | -1.334 | 0.0092513 |
| CYCS | ENSG00000172115 | 120.858 | 161.119 | -1.333 | 0.042812 |
| RNF10 | ENSG00000022840 | 145.486 | 193.839 | -1.332 | 0.02664 |
| RPN1 | ENSG00000163902 | 269.080 | 357.560 | -1.329 | 0.0028835 |
| GPX1 | ENSG00000233276 | 416.619 | 553.506 | -1.329 | 0.0002113 |
| TMED9 | ENSG00000184840 | 123.823 | 164.094 | -1.325 | 0.045821 |
| CAPZB | ENSG00000077549 | 164.071 | 217.263 | -1.324 | 0.021971 |
| PRDX1 | ENSG00000117450 | 486.397 | 642.865 | -1.322 | 9.17E-05 |
| COTL1 | ENSG00000103187 | 365.083 | 481.374 | -1.319 | 0.0008027 |
| CEBPB | ENSG00000172216 | 179.235 | 236.225 | -1.318 | 0.019094 |
| MT-CO2 | ENSG00000198712 | 2533.346 | 3337.272 | -1.317 | 1.32E-18 |
| COL4A1 | ENSG00000187498 | 130.892 | 172.397 | -1.317 | 0.045852 |
| FOLR1 | ENSG00000110195 | 2119.692 | 2791.450 | -1.317 | 9.04E-16 |
| RPS27 | ENSG00000177954 | 1022.163 | 1344.973 | -1.316 | 2.73E-08 |
| COX7C | ENSG00000127184 | 203.863 | 268.201 | -1.316 | 0.013175 |
| PEBP1 | ENSG00000089220 | 227.578 | 299.310 | -1.315 | 0.0089083 |
| HSD17B10 | ENSG00000072506 | 215.834 | 283.694 | -1.314 | 0.011083 |
| ZNF622 | ENSG00000173545 | 130.322 | 171.282 | -1.314 | 0.0485 |
| NUDC | ENSG00000090273 | 182.655 | 239.943 | -1.314 | 0.019787 |
| ADH5 | ENSG00000197894 | 143.662 | 188.633 | -1.313 | 0.039182 |
| HLA-B | ENSG00000234745 | 354.593 | 465.510 | -1.313 | 0.0012053 |
| SUB1 | ENSG00000113387 | 220.281 | 289.147 | -1.313 | 0.010771 |
| RPL39 | ENSG00000198918 | 1021.821 | 1339.891 | -1.311 | 4.54E-08 |
| GIPC1 | ENSG00000123159 | 154.835 | 202.638 | -1.309 | 0.035004 |
| SLC38A1 | ENSG00000111371 | 875.081 | 1143.450 | -1.307 | 6.46E-07 |
| PGAM1 | ENSG00000171314 | 372.380 | 486.456 | -1.306 | 0.0011877 |
| PARK7 | ENSG00000116288 | 202.266 | 264.111 | -1.306 | 0.017129 |
| YWHAQ | ENSG00000134308 | 284.131 | 370.946 | -1.306 | 0.0047518 |
| ANXA1 | ENSG00000135046 | 168.061 | 219.370 | -1.305 | 0.030059 |
| PSMC3 | ENSG00000165916 | 350.489 | 457.330 | -1.305 | 0.0017612 |
| ARL6IP1 | ENSG00000170540 | 170.342 | 222.096 | -1.304 | 0.029835 |
| PSMA4 | ENSG00000041357 | 205.117 | 267.210 | -1.303 | 0.017594 |
| EPRS | ENSG00000136628 | 346.042 | 450.638 | -1.302 | 0.0020797 |
| BCAP31 | ENSG00000185825 | 156.089 | 203.010 | -1.301 | 0.039907 |
| NPNT | ENSG00000168743 | 136.707 | 105.099 | 1.301 | 0.017081 |
| BRD3 | ENSG00000169925 | 144.688 | 110.800 | 1.306 | 0.013051 |
| IGF1R | ENSG00000140443 | 256.310 | 196.193 | 1.306 | 0.0009383 |
| BEGAIN | ENSG00000183092 | 113.561 | 86.880 | 1.307 | 0.027425 |
| FGD1 | ENSG00000102302 | 114.815 | 87.748 | 1.308 | 0.026121 |
| LGR4 | ENSG00000205213 | 134.084 | 102.373 | 1.310 | 0.015916 |
| CSPG4 | ENSG00000173546 | 293.024 | 223.708 | 1.310 | 0.000364 |
| PLEC | ENSG00000178209 | 2009.666 | 1532.490 | 1.311 | 6.94E-21 |
| FOXK1 | ENSG00000164916 | 167.149 | 127.408 | 1.312 | 0.0068365 |
| H6PD | ENSG00000049239 | 125.989 | 95.928 | 1.313 | 0.018487 |
| INCENP | ENSG00000149503 | 173.990 | 132.366 | 1.314 | 0.005519 |
| CPNE7 | ENSG00000178773 | 116.982 | 88.987 | 1.315 | 0.022845 |
| MYH10 | ENSG00000133026 | 105.466 | 80.188 | 1.315 | 0.030452 |
| NLGN2 | ENSG00000169992 | 95.090 | 72.256 | 1.316 | 0.039544 |
| ZNF24 | ENSG00000172466 | 93.950 | 71.388 | 1.316 | 0.040736 |
| TTYH3 | ENSG00000136295 | 96.344 | 73.123 | 1.318 | 0.037619 |
| VGLL3 | ENSG00000206538 | 119.604 | 90.598 | 1.320 | 0.019836 |
| KNTC1 | ENSG00000184445 | 123.823 | 93.697 | 1.322 | 0.017446 |
| PRRC2B | ENSG00000130723 | 983.056 | 741.396 | 1.326 | 1.34E-11 |
| RAI1 | ENSG00000108557 | 130.892 | 98.654 | 1.327 | 0.013453 |
| MED25 | ENSG00000104973 | 93.722 | 70.397 | 1.331 | 0.034731 |
| TONSL | ENSG00000160949 | 85.969 | 64.572 | 1.331 | 0.043137 |
| UBXN7 | ENSG00000163960 | 83.232 | 62.465 | 1.332 | 0.046099 |
| VPS51 | ENSG00000149823 | 142.293 | 106.710 | 1.333 | 0.0089707 |
| H19 | ENSG00000130600 | 390.281 | 291.749 | 1.338 | 1.26E-05 |
| SETD1B | ENSG00000139718 | 130.664 | 97.663 | 1.338 | 0.011503 |
| MEGF8 | ENSG00000105429 | 107.176 | 80.064 | 1.339 | 0.021902 |
| KCNN4 | ENSG00000104783 | 102.045 | 76.222 | 1.339 | 0.02527 |
| WBP7 | ENSG00000105663 | 123.138 | 91.962 | 1.339 | 0.013942 |
| MLLT6 | ENSG00000108292 | 328.255 | 244.653 | 1.342 | 5.42E-05 |
| GLTSCR2 | ENSG00000105373 | 578.637 | 431.179 | 1.342 | 8.18E-08 |
| ESRP2 | ENSG00000103067 | 82.890 | 61.721 | 1.343 | 0.042005 |
| FAM127A | ENSG00000134590 | 77.418 | 57.631 | 1.343 | 0.049235 |
| RPS2P46 | ENSG00000189343 | 146.968 | 109.313 | 1.344 | 0.0066116 |
| ARID5B | ENSG00000150347 | 102.843 | 76.470 | 1.345 | 0.022984 |
| ARHGAP23 | ENSG00000225485 | 417.759 | 310.216 | 1.347 | 4.24E-06 |
| TCIRG1 | ENSG00000110719 | 83.461 | 61.969 | 1.347 | 0.039775 |
| DAB2IP | ENSG00000136848 | 141.723 | 105.099 | 1.348 | 0.0071852 |
| DST | ENSG00000151914 | 116.640 | 86.385 | 1.350 | 0.014401 |
| PKD1 | ENSG00000008710 | 149.818 | 110.924 | 1.351 | 0.0055094 |
| SF3A2 | ENSG00000104897 | 76.962 | 56.887 | 1.353 | 0.045716 |
| SOGA1 | ENSG00000149639 | 314.117 | 231.888 | 1.355 | 5.11E-05 |
| TPR | ENSG00000047410 | 177.867 | 131.126 | 1.356 | 0.0022233 |
| ADCY6 | ENSG00000174233 | 235.559 | 173.513 | 1.358 | 0.0004192 |
| SYNE2 | ENSG00000054654 | 147.538 | 108.446 | 1.360 | 0.0049975 |
| RERE | ENSG00000142599 | 96.344 | 70.645 | 1.364 | 0.02245 |
| TCF3 | ENSG00000071564 | 216.747 | 158.888 | 1.364 | 0.0006121 |
| JUND | ENSG00000130522 | 283.105 | 207.472 | 1.365 | 8.90E-05 |
| IRS2 | ENSG00000185950 | 221.991 | 162.482 | 1.366 | 0.0004994 |
| C5orf42 | ENSG00000197603 | 161.220 | 117.989 | 1.366 | 0.0030035 |
| ITGB4 | ENSG00000132470 | 315.143 | 230.524 | 1.367 | 3.26E-05 |
| POLD1 | ENSG00000062822 | 177.183 | 129.391 | 1.369 | 0.0017603 |
| COL12A1 | ENSG00000111799 | 468.838 | 341.325 | 1.374 | 2.92E-07 |
| WTIP | ENSG00000142279 | 83.461 | 60.730 | 1.374 | 0.030291 |
| OBSCN | ENSG00000154358 | 391.079 | 284.313 | 1.376 | 2.60E-06 |
| AHNAK | ENSG00000124942 | 2413.286 | 1754.339 | 1.376 | 1.47E-31 |
| FBRSL1 | ENSG00000112787 | 106.036 | 76.965 | 1.378 | 0.014014 |
| ZKSCAN1 | ENSG00000106261 | 100.449 | 72.875 | 1.378 | 0.01666 |
| LTBP3 | ENSG00000168056 | 101.589 | 73.619 | 1.380 | 0.015758 |
| PIEZO1 | ENSG00000103335 | 631.541 | 457.578 | 1.380 | 1.70E-09 |
| BTAF1 | ENSG00000095564 | 115.157 | 83.162 | 1.385 | 0.0095221 |
| SPEN | ENSG00000065526 | 259.161 | 187.022 | 1.386 | 9.76E-05 |
| WDR90 | ENSG00000161996 | 139.785 | 100.514 | 1.391 | 0.0038935 |
| MTA1 | ENSG00000182979 | 84.487 | 60.730 | 1.391 | 0.024701 |
| ZC3H4 | ENSG00000130749 | 88.021 | 63.208 | 1.393 | 0.021564 |
| LDB1 | ENSG00000198728 | 84.829 | 60.853 | 1.394 | 0.023731 |
| AGRN | ENSG00000188157 | 1276.650 | 914.909 | 1.395 | 1.38E-18 |
| SLC7A6 | ENSG00000103064 | 68.410 | 48.955 | 1.397 | 0.041076 |
| GPRIN2 | ENSG00000204175 | 81.294 | 58.127 | 1.399 | 0.025664 |
| PRR12 | ENSG00000126464 | 142.179 | 101.505 | 1.401 | 0.0030601 |
| PC | ENSG00000173599 | 207.055 | 147.734 | 1.402 | 0.0003448 |
| MYLK | ENSG00000065534 | 231.341 | 164.961 | 1.402 | 0.0001515 |
| MYRF | ENSG00000124920 | 64.534 | 45.857 | 1.407 | 0.043664 |
| LRRC37A4P | ENSG00000214425 | 61.455 | 43.626 | 1.409 | 0.048478 |
| MLL2 | ENSG00000167548 | 398.718 | 282.578 | 1.411 | 4.51E-07 |
| LENG8 | ENSG00000167615 | 220.851 | 156.409 | 1.412 | 0.000169 |
| SAPCD2 | ENSG00000186193 | 74.795 | 52.921 | 1.413 | 0.028272 |
| SLC5A3 | ENSG00000198743 | 105.352 | 74.239 | 1.419 | 0.008595 |
| MYC | ENSG00000136997 | 303.628 | 213.297 | 1.423 | 7.03E-06 |
| SOGA2 | ENSG00000168502 | 103.870 | 72.132 | 1.440 | 0.0070201 |
| LAMC3 | ENSG00000050555 | 56.210 | 38.916 | 1.444 | 0.045893 |
| CCAT1 | ENSG00000247844 | 218.685 | 151.328 | 1.445 | 8.07E-05 |
| NFAT5 | ENSG00000102908 | 159.966 | 110.676 | 1.445 | 0.000744 |
| PFAS | ENSG00000178921 | 160.992 | 111.296 | 1.447 | 0.0007 |
| PPARA | ENSG00000186951 | 59.745 | 41.271 | 1.448 | 0.038624 |
| CLUH | ENSG00000132361 | 262.809 | 181.445 | 1.448 | 1.40E-05 |
| AGO2 | ENSG00000123908 | 75.251 | 51.930 | 1.449 | 0.020007 |
| PTPRS | ENSG00000105426 | 183.454 | 126.417 | 1.451 | 0.0002689 |
| ZSWIM4 | ENSG00000132003 | 92.810 | 63.952 | 1.451 | 0.0095513 |
| RP11-46C20.1 | ENSG00000250337 | 66.244 | 45.609 | 1.452 | 0.028282 |
| PTPN14 | ENSG00000152104 | 89.960 | 61.845 | 1.455 | 0.010342 |
| BRWD3 | ENSG00000165288 | 52.904 | 36.314 | 1.457 | 0.048512 |
| THBS1 | ENSG00000137801 | 190.865 | 131.002 | 1.457 | 0.0001782 |
| XRCC2 | ENSG00000196584 | 83.004 | 56.763 | 1.462 | 0.012749 |
| RECQL4 | ENSG00000160957 | 131.006 | 89.483 | 1.464 | 0.0017071 |
| RBM15 | ENSG00000162775 | 74.339 | 50.567 | 1.470 | 0.017147 |
| DDR1 | ENSG00000204580 | 92.354 | 62.713 | 1.473 | 0.0076716 |
| ABCA2 | ENSG00000107331 | 389.711 | 263.740 | 1.478 | 3.47E-08 |
| TICRR | ENSG00000140534 | 91.784 | 62.093 | 1.478 | 0.0073967 |
| CITED4 | ENSG00000179862 | 249.812 | 168.307 | 1.484 | 8.34E-06 |
| MUC16 | ENSG00000181143 | 66.472 | 44.618 | 1.490 | 0.020567 |
| NR2F1 | ENSG00000175745 | 55.526 | 37.181 | 1.493 | 0.03344 |
| MAN2A2 | ENSG00000196547 | 96.002 | 63.952 | 1.501 | 0.0047223 |
| FASN | ENSG00000169710 | 434.177 | 289.147 | 1.502 | 1.83E-09 |
| GPSM1 | ENSG00000160360 | 109.913 | 73.123 | 1.503 | 0.0024385 |
| GOLGA8A | ENSG00000175265 | 153.923 | 102.125 | 1.507 | 0.0003111 |
| LAMA3 | ENSG00000053747 | 58.491 | 38.793 | 1.508 | 0.026123 |
| LRP1 | ENSG00000123384 | 219.255 | 145.255 | 1.509 | 1.59E-05 |
| FGFR3 | ENSG00000068078 | 75.821 | 49.947 | 1.518 | 0.010291 |
| SACS | ENSG00000151835 | 76.734 | 50.443 | 1.521 | 0.0095541 |
| LTBP4 | ENSG00000090006 | 174.788 | 114.395 | 1.528 | 8.00E-05 |
| MEX3D | ENSG00000181588 | 78.102 | 50.938 | 1.533 | 0.0079603 |
| HERC2P2 | ENSG00000140181 | 62.139 | 40.528 | 1.533 | 0.01793 |
| CELSR2 | ENSG00000143126 | 130.094 | 84.649 | 1.537 | 0.0005817 |
| KIAA1147 | ENSG00000257093 | 44.923 | 29.125 | 1.542 | 0.041864 |
| LAMA5 | ENSG00000130702 | 1329.782 | 858.765 | 1.548 | 6.61E-29 |
| MMP28 | ENSG00000129270 | 40.932 | 26.399 | 1.551 | 0.049858 |
| MED12 | ENSG00000184634 | 64.078 | 41.271 | 1.553 | 0.013904 |
| TCAM1P | ENSG00000240280 | 49.483 | 31.728 | 1.560 | 0.029319 |
| FOXE1 | ENSG00000178919 | 173.762 | 110.800 | 1.568 | 3.72E-05 |
| TMEM132A | ENSG00000006118 | 64.762 | 41.271 | 1.569 | 0.011726 |
| ZNF703 | ENSG00000183779 | 40.704 | 25.903 | 1.571 | 0.04517 |
| ADAMTSL4 | ENSG00000143382 | 132.944 | 84.526 | 1.573 | 0.0002879 |
| SIPA1L3 | ENSG00000105738 | 85.399 | 54.037 | 1.580 | 0.0033843 |
| C9orf172 | ENSG00000232434 | 46.633 | 29.373 | 1.588 | 0.029064 |
| B4GALNT2 | ENSG00000167080 | 52.676 | 32.967 | 1.598 | 0.019012 |
| C19orf71 | ENSG00000183397 | 65.674 | 40.899 | 1.606 | 0.008274 |
| GPRIN3 | ENSG00000185477 | 53.474 | 33.215 | 1.610 | 0.016702 |
| EPPK1 | ENSG00000227184 | 342.507 | 210.942 | 1.624 | 8.08E-10 |
| NBEAL2 | ENSG00000160796 | 53.132 | 32.596 | 1.630 | 0.014901 |
| GOLGA2B | ENSG00000238105 | 54.842 | 33.091 | 1.657 | 0.011086 |
| NOTCH1 | ENSG00000148400 | 149.476 | 89.979 | 1.661 | 2.56E-05 |
| LRRK1 | ENSG00000154237 | 93.038 | 54.904 | 1.695 | 0.0006196 |
| CTD-2653M23.2 | ENSG00000248044 | 30.443 | 17.847 | 1.706 | 0.04802 |
| DNHD1 | ENSG00000179532 | 30.101 | 17.599 | 1.710 | 0.048399 |
| FBLN7 | ENSG00000144152 | 85.741 | 49.823 | 1.721 | 0.0007751 |
| PTGS1 | ENSG00000095303 | 117.666 | 67.422 | 1.745 | 5.87E-05 |
| CTC-295J13.3 | ENSG00000248109 | 32.381 | 18.343 | 1.765 | 0.032227 |
| PTPRQ | ENSG00000139304 | 28.162 | 15.740 | 1.789 | 0.041939 |
| GLP2R | ENSG00000065325 | 26.794 | 14.873 | 1.802 | 0.045235 |
| ENGASE | ENSG00000167280 | 28.162 | 15.244 | 1.847 | 0.033927 |
| IRS1 | ENSG00000169047 | 54.272 | 28.630 | 1.896 | 0.0023662 |
| RP11-206L10.1 | ENSG00000224956 | 26.110 | 13.633 | 1.915 | 0.032796 |
| HSPG2 | ENSG00000142798 | 510.455 | 262.128 | 1.947 | 6.22E-22 |
| RNF150 | ENSG00000170153 | 21.891 | 10.163 | 2.154 | 0.026489 |
| TPTEP1 | ENSG00000100181 | 18.927 | 2.727 | 6.941 | 0.0001713 |

*Genes with |fold-change|>=1.3 and *P*<0.05.

**Table S7.** KEGG pathways identified by GSEA.

| **Gene Set** | **Description** | **Size** | **Leading Edge Number** | **ES** | **NES** | **P Value** | **FDR** |
| --- | --- | --- | --- | --- | --- | --- | --- |
| hsa01100 | Metabolic pathways | 71 | 44 | -0.35959 | -2.6588 | <2.2e-16 | <2.2e-16 |
| hsa01230 | Biosynthesis of amino acids | 12 | 9 | -0.65586 | -2.5013 | <2.2e-16 | 0.0005801 |
| hsa00260 | Glycine, serine and threonine metabolism | 6 | 5 | -0.72049 | -2.0499 | 0.0017513 | 0.017016 |
| hsa04141 | Protein processing in endoplasmic reticulum | 18 | 17 | -0.38662 | -1.7232 | 0.037097 | 0.099484 |
| hsa00270 | Cysteine and methionine metabolism | 6 | 4 | -0.62291 | -1.796 | 0.020942 | 0.06688 |
| hsa05202 | Transcriptional misregulation in cancer | 10 | 5 | -0.50662 | -1.8125 | 0.0099668 | 0.063592 |
| hsa05208 | Chemical carcinogenesis | 23 | 22 | -0.42341 | -2.1336 | <2.2e-16 | 0.012568 |
| hsa00190 | Oxidative phosphorylation | 24 | 23 | -0.37666 | -1.9317 | 0.0016313 | 0.032968 |
| hsa01240 | Biosynthesis of cofactors | 9 | 9 | -0.57219 | -1.9305 | 0.0087873 | 0.030611 |
| hsa04150 | **mTOR signaling pathway*** | 8 | **5*** | -0.50226 | -1.6195 | 0.038917 | 0.15087 |
| hsa04510 | Focal adhesion | 9 | 8 | 0.50421 | 1.7838 | 0.011765 | 0.27821 |
| hsa04512 | ECM-receptor interaction | 9 | 9 | 0.61497 | 2.1259 | <2.2e-16 | 0.054962 |

*Genes involved include: EIF4EBP1, SESN2, GRB10, LAMTOR5, and RRAGC.
